# The efficacy of complementary and alternative medicine non-pharmacological interventions in improving disease activity and inflammation in ankylosing spondylitis: a network meta-analysis

**DOI:** 10.3389/fmed.2026.1824982

**Published:** 2026-08-03

**Authors:** Qianyiqi He, Long Qu, Mengling Sun, Huiyu Li, Liang Sun, Wei Cao

**Affiliations:** 1 Heilongjiang Academy of Traditional Chinese Medicine, Harbin, China; 2 Heilongjiang Provincial Hospital of Traditional Chinese Medicine, Harbin, China

**Keywords:** complementary and alternative medicine, CAM, ankylosing spondylitis, AS, efficacy, network meta-analysis, NMA

## Abstract

**Objective:**

Ankylosing spondylitis (AS), a chronic progressive autoimmune disease primarily affecting the axial joints, frequently causes symptoms such as pain, stiffness, and functional limitations. In recent years, complementary and alternative medicine (CAM) non-pharmacological therapies have gained increasing recognition within the academic community for their potential efficacy in alleviating AS symptoms. This study aims to evaluate the effectiveness of CAM therapies in relieving AS symptoms.

**Methods:**

This systematic review was prospectively registered in the International Prospective Register of Systematic Reviews (PROSPERO; registration number: CRD420261323792). A comprehensive search of English and Chinese databases was conducted from their inception until 1 January 2026. Search criteria focused on prospective controlled trials of CAM therapies for AS patients. Statistical analysis employed mean values and standard deviations. Additionally, study quality and potential bias were assessed.

**Results:**

Thirty-one randomized controlled trials involving 2,459 patients with ankylosing spondylitis and 15 complementary and alternative medicine interventions were included. The network meta-analysis (NMA) revealed that, compared with conventional therapy, AI (MD = −1.81, 95% CI [−2.90, −0.73]), Acu (MD = −1.63, 95% CI [−2.85, −0.41]), Moxibustion (MD = −1.51, 95% CI [−2.58, −0.44]), and Moxibustion + Cupping (MD = −1.05, 95% CI [−2.07, −0.03]) demonstrated statistically significant improvements in BASDAI scores. Regarding functional status (BASFI), several CAM interventions showed favorable trends, although further evaluation is required. For pain relief (VAS), IH (MD = −1.75, 95% CI [−2.98, −0.52]), AA (MD = −1.37, 95% CI [−2.46, −0.28]), Acu (MD = −1.05, 95% CI [−2.03, −0.07]), and Moxibustion (MD = −0.76, 95% CI [−1.46, −0.06]) demonstrated statistically significant improvements compared with conventional therapy. Regarding inflammatory markers, Moxibustion (CRP: MD = −40.69, 95% CI [−76.83, −4.55]) showed a statistically significant reduction in C-reactive protein levels compared with conventional therapy. SUCRA probability ranking suggested that some CAM interventions had higher probabilities of achieving favorable outcomes; however, these rankings should be interpreted cautiously because they represent relative probabilities rather than definitive evidence of clinical superiority. The observed differences among interventions should primarily be considered together with effect estimates, confidence intervals, and clinical relevance to determine their true clinical effectiveness and safety.

**Conclusion:**

Moxibustion combined with cupping showed a favorable probability of improving pain outcomes; however, this finding requires confirmation due to limited certainty of evidence. Several CAM interventions demonstrated potential benefits in improving disease-related outcomes, including disease activity, pain, and inflammatory markers. However, the safety profile of CAM interventions should be interpreted cautiously because adverse events were incompletely reported among the included studies. Overall, the available evidence does not allow definitive conclusions regarding the safety of CAM therapies in patients with ankylosing spondylitis.

**Systematic review registration:**

CRD420261323792; https://doi.org/10.37766/inplasy2025.12.0043.

## 1 Introduction

Ankylosing spondylitis (AS) is an autoimmune disorder primarily characterized by chronic inflammation of the sacroiliac joints and spine, predominantly affecting young to middle-aged males (1). Its clinical manifestations include low back pain, morning stiffness, and restricted mobility. In severe cases, it may lead to spinal ankylosis and deformity, significantly impairing patients' quality of life (2). The global prevalence is approximately 0.1%-0.3%, with China reporting around 0.3% (1). Conventional Western medical treatment remains the mainstream approach, primarily comprising non-steroidal anti-inflammatory drugs, disease-modifying anti-rheumatic drugs, and biologics. Its advantages lie in a relatively rapid onset of action, effective relief of pain and stiffness, and significant improvement in joint function. Notably, the application of biologics can block inflammatory responses at their source, marking a milestone in controlling disease progression and preventing joint deformities. They currently represent potentially favorable means of inhibiting disease progression (3). However, Western medical treatment also faces challenges. For instance, long-term use of NSAIDs may cause gastrointestinal reactions and impair liver and kidney function; disease-modifying antirheumatic drugs (DMARDs) and biologics may increase infection risks, and some patients cannot afford biologic therapy due to financial constraints (3–5).

Conventional Western medical treatment remains the mainstream approach. However, Western medical treatment also faces challenges.

Recent real-world evidence has further highlighted the importance of non-pharmacological interventions in the long-term management of ankylosing spondylitis (AS). Exercise therapy, physiotherapy, acupuncture, and other complementary rehabilitation approaches have demonstrated significant benefits in improving pain, physical function, spinal mobility, and quality of life among patients with axial spondyloarthritis. The 2022 ASAS-EULAR recommendations emphasize that regular exercise and patient education should constitute the foundation of AS management and should be maintained throughout the disease course, regardless of pharmacological treatment status. Furthermore, a systematic literature review supporting the ASAS-EULAR recommendations confirmed that structured exercise programs consistently improve disease activity, physical function, and mobility outcomes, with minimal safety concerns (6).

Compared with pharmacological therapies, non-pharmacological interventions possess several unique clinical advantages. Although non-steroidal anti-inflammatory drugs (NSAIDs) and biologic agents can rapidly suppress inflammation and alleviate symptoms, their long-term use may be associated with gastrointestinal adverse events, increased infection risk, immunosuppression, and a substantial economic burden. In contrast, exercise-based rehabilitation and complementary medicine interventions are generally associated with fewer adverse effects, better patient adherence, and lower treatment costs. A recent meta-analysis demonstrated that physiotherapy-based interventions significantly improved functional outcomes and disease-related symptoms in patients with AS, supporting their role as effective adjunctive or complementary treatment strategies (7).

Moreover, real-world observational studies indicate that many patients continue to experience residual pain, functional limitations, and reduced quality of life despite receiving biologic therapy, highlighting the need for comprehensive multimodal management approaches. Consequently, non-pharmacological interventions are increasingly recognized not merely as adjunctive therapies but as essential components of integrated AS management. However, despite the growing body of evidence, direct comparisons among different complementary and alternative medicine (CAM) interventions remain limited, and the relative efficacy of these approaches for improving disease activity, pain, function, and inflammatory markers has not been comprehensively established. Therefore, a network meta-analysis is warranted to compare multiple CAM therapies simultaneously and identify potentially favorable treatment strategies for patients with AS (8).

Complementary and alternative medicine (CAM) encompasses diverse practices outside mainstream healthcare systems, including acupuncture, massage therapy, moxibustion, and cupping. These therapies promote health holistically and naturally, emphasizing prevention and individualization, and are widely applied in the clinical management of AS (9, 10).

Network meta-analysis (NMA), an innovative meta-analytic method, enables more intuitive and comprehensive comparisons of the efficacy of multiple interventions, whereas traditional meta-analysis is limited to pairwise comparisons. Currently, NMAs specifically addressing CAM therapies for AS remain scarce. This NMA builds upon existing research concerning complementary and alternative medicine (CAM) to evaluate the efficacy and safety of different external CAM therapies for patients with ankylosing spondylitis (AS). It focuses on pain relief, functional improvement, and inflammatory markers to determine a potentially favorable treatment regimen.

## 2 Materials and methods

### 2.1 Protocol and registration

This meta-analysis was conducted in strict adherence to the Preferred Reporting Items for Systematic Reviews and Meta-Analyses (PRISMA) guidelines. The study protocol was prospectively registered in the International Prospective Register of Systematic Reviews (PROSPERO) under registration number CRD420261323792.

### 2.2 Search strategy

The search strategy was developed in accordance with the Preferred Reporting Items for Systematic Reviews and Meta-Analyses (PRISMA 2020) statement and the recommendations of the Cochrane Handbook for Systematic Reviews of Interventions. In addition, the search terms and retrieval framework were informed by previously published systematic reviews and network meta-analyses investigating complementary and alternative medicine (CAM) interventions for ankylosing spondylitis (AS), thereby ensuring the comprehensiveness and reproducibility of the search strategy (9–11). The core methodology for literature screening and data extraction involved combining Medical Subject Headings (MeSH) with free-text terms for retrieval. Literature screening and data extraction were performed independently by two reviewers (He QYQ and Sun ML). Any discrepancies were resolved through consultation with a third reviewer (corresponding author). The search timeframe spanned from the inception of each database to 1 January 2026, aiming to collect all RCTs focusing on ankylosing spondylitis (AS) and external treatments involving complementary and alternative medicine (CAM). The databases searched included the Cochrane Library, PubMed, Embase, Web of Science, China National Knowledge Infrastructure (CNKI) Full-text Database of Chinese Journals, Wanfang Academic Journal Full-text Database, VIP Database of Chinese Science and Technology Journals, and China Biomedical Literature Database. Additionally, manual searches were conducted to supplement electronic database results; search terms are detailed in the Supplementary materials.

### 2.3 Inclusion and exclusion criteria

Study Type: This meta-analysis exclusively included randomized controlled trials (RCTs) published in Chinese or English. Where the same study appeared in multiple publications, these were consolidated into a single entry for analysis.

Study Participants: Eligible participants must meet the 1984 American College of Rheumatology (ACR) diagnostic criteria for ankylosing spondylitis (AS) (12), or be diagnosed according to the 2010 AS diagnostic guidelines proposed by the Chinese Medical Association Rheumatology Branch (CMA) (12).

Interventions: All complementary and alternative medicine (CAM) therapies for AS patients were included. The intervention group required at least one CAM therapy, used alone or in combination. No restrictions were imposed on materials, treatment sites, duration, or frequency. The control group received oral medication or conventional treatments, including both Western and traditional Chinese medicine approaches, used alone or in combination.

Exclusion Criteria: Studies were excluded based on the following criteria: a. Studies lacking a clear diagnosis of ankylosing spondylitis (AS) or involving other comorbid conditions; b. Studies not employing complementary and alternative medicine (CAM) as a treatment modality; c. Non-journal publications, including conference papers, abstracts, and theses; d. Studies not being randomized controlled trials (RCTs); e. Studies where the full text was unavailable; f. Studies incorporating Chinese herbal medicine interventions; g. Studies lacking clear outcome measures or baseline data; h. Studies where data were not presented as mean and standard deviation; i. Duplicate publications of the same study; j. Meta-analysis articles; k. Prospective-retrospective studies within the same patient cohort.

Outcome measure types: All studies must include at least one of the following outcome measures. The primary objective is to evaluate improvements in functional status, pain, and inflammatory markers in AS patients following complementary and alternative medicine (CAM) treatment. The outcome measures employed are as follows: a. Bath Ankylosing Spondylitis Disease Activity Index (BASDAI) to assess subjective symptom severity; b. Bath Ankylosing Spondylitis Functional Index (BASFI) to evaluate objective functional capacity; c. Visual Analog Scale (VAS) using a 10-point scoring system to assess pain intensity; d. C-reactive protein (CRP) to monitor systemic inflammatory activity; e. Erythrocyte sedimentation rate (ESR) to monitor systemic inflammatory activity. Specific criteria are detailed in Table 1.

**Table 1:** PICOS criteria.

Criteria	Explanation
Population	Adults (≥18 yrs) with spondylitis, ankylosing (Unilateral grade III-IV sacroiliitis, or bilateral grade II-III sacroiliitis)
Intervention	CAM
Comparison	Conventional therapy
Outcome	BASDAI, BASFI, VAS, CRP, ESR
Study design	RCTs

### 2.4 Study screening and data extraction

Two researchers (He QYQ and Sun ML) independently screened the literature and extracted relevant data. Following preliminary extraction, findings were cross-checked to ensure accuracy and consistency. Disagreements were resolved through discussion, with consultation of an independent third party (corresponding author) where necessary. Data extracted included: a. study background information; b. baseline characteristics of participants and treatment regimens employed; c. key elements for assessing risk of bias; d. outcome measures and associated data.

### 2.5 Risk of bias in included studies

Risk of bias assessments were conducted independently by two researchers (He QYQ and Sun ML). Disagreements were resolved by a third reviewer (corresponding author). Data were cross-validated post-extraction. In cases of disagreement, a third researcher was consulted to make a final determination. Risk of bias assessments for randomized controlled trials (RCTs) employed the tool recommended in Chapter 5.1.0 of the Cochrane Handbook for Systematic Reviews (13).

### 2.6 Statistical analysis

This network meta-analysis (NMA) employed Stata 18.0 software (Stata Corp., College Station, Texas, USA), specifically utilizing the mvmeta package for data analysis. Statistical analysis utilized the mean and standard deviation of collected data. Assessment of study quality and risk of bias was conducted using the ROB 2.0 tool. Given that all data in this study were measured, the mean difference (MD) and 95% confidence interval (CI) were selected as the primary statistical measures. Heterogeneity between studies was assessed by comparing prediction interval plots, with local inconsistency assessed using node-splitting methods when closed loops were available. Clinical heterogeneity among included studies was carefully considered before conducting network meta-analysis. Potential sources of heterogeneity, including differences in control interventions, CAM treatment protocols, treatment frequency, treatment duration, concomitant conventional medications, and baseline patient characteristics, were assessed during data extraction and network construction. When substantial clinical differences were identified, the findings were interpreted cautiously, and potential impacts on the transitivity assumption were considered. Similarity between studies was assessed by comparing key clinical and methodological characteristics before conducting network meta-analysis. The transitivity assumption, which represents the fundamental prerequisite for valid indirect comparisons in network meta-analysis, was carefully considered by evaluating potential effect modifiers across treatment comparisons. Specifically, baseline characteristics, including patient age, disease duration, baseline disease activity, intervention duration, outcome measurement methods, and concomitant conventional treatments, were examined to determine whether meaningful differences existed among the included studies. Although variations were observed across some characteristics, the overall clinical and methodological similarity among included trials was considered acceptable for performing indirect comparisons. The significance level for network meta-analyses (NMAs) was set at α = 0.05, with statistical significance determined when the 95% CI did not include zero. Network consistency was assessed using both global and local approaches. For closed-loop comparisons, local inconsistency was evaluated using node-splitting methods to compare direct and indirect evidence. When inconsistency was detected, potential sources were explored by examining differences in clinical characteristics, intervention protocols, and study methodology. Considering the anticipated clinical and methodological heterogeneity across studies—including variations in participant characteristics, intervention protocols, treatment duration, and outcome assessment methods—a random-effects model was applied for all network meta-analyses regardless of the magnitude of statistical heterogeneity. Subgroup analyses were planned a priori to explore potential sources of heterogeneity when sufficient data were available. Pre-specified factors included intervention characteristics, treatment duration, and other clinically relevant variables. Meta-regression was considered as an additional approach to investigate potential effect modifiers; however, it was not performed because the number of studies available for individual comparisons was insufficient to provide reliable estimates and avoid model overfitting. Therefore, subgroup analyses were only conducted for variables with adequate data availability, and the results were interpreted as exploratory rather than confirmatory. The Surface Under Cumulative Ranking Curve (SUCRA) is employed to probabilistically rank efficacy measures of interventions. SUCRA provides a probabilistic assessment of the relative efficacy rankings of different interventions based on current evidence networks, reflecting only “relative likelihood” rather than “absolute clinical superiority.” As an exploratory analytical tool, its results should be interpreted in conjunction with statistical significance from pairwise comparisons, study quality, sample size, publication bias, and other factors; it should not be used alone as the definitive criterion for selecting the superior clinical intervention. Concurrently, Stata 18.0 software was utilized to generate a publication bias funnel plot (comparison adjustment plot). Literature quality assessment and risk of bias analysis were conducted using the ROB 2.0 tool.

Multi-arm trials necessitate subgroup consolidation during data processing and analysis, either by adopting methods from two-arm trials or by stratifying and analyzing according to distinct interventions. For multi-arm trials, subgroups receiving identical interventions (e.g., acupuncture with varying durations) were pooled to avoid duplicate calculations. Where different interventions were involved, data from multi-arm studies were divided into independent comparison groups. This approach was employed to increase the sample size and enhance statistical power. The use of a random-effects model was pre-specified to account for between-study variability and is consistent with current recommendations for network meta-analysis involving diverse non-pharmacological interventions.

### 2.7 Assessment of confidence in evidence

Network geometry was evaluated to describe the structure of available evidence. Network plots were generated for each outcome, with nodes representing individual interventions and edges representing direct comparisons between interventions. The size of each node reflected the number of participants receiving the corresponding intervention, while the thickness of connecting lines represented the number of direct comparisons available between interventions. This approach allowed visualization of the distribution and availability of direct evidence within the network.

The assumption of consistency between direct and indirect evidence was assessed using appropriate methods for network meta-analysis. Global inconsistency was assessed through overall model evaluation. A *P*-value < 0.05 was considered indicative of statistically significant inconsistency.

Clinical interpretation of treatment effects was guided by minimal clinically important differences (MCIDs) within the CINeMA framework. For BASDAI, BASFI, and pain VAS outcomes, ranges of equivalence were defined according to published MCID estimates. Specifically, the MCID was set at 1.0 point for BASDAI, 0.7 points for BASFI, and 10 mm for pain VAS. These thresholds were used to judge whether treatment effects were clinically important when assessing the CINeMA domain of clinical relevance.

No validated and widely accepted MCID has been established for CRP (mg/L) or ESR (mm/h) in patients with ankylosing spondylitis. Therefore, MCID-based thresholds were not applied to these laboratory outcomes, and the results were interpreted descriptively rather than through predefined ranges of equivalence.

Statistical significance and clinical relevance were interpreted as separate concepts in this study. Statistical significance was determined according to the 95% confidence intervals of treatment effects, whereas clinical relevance was assessed according to predefined MCID thresholds when available. Therefore, statistically significant effects were not automatically considered clinically meaningful unless they exceeded the established MCID values.

## 3 Results

### 3.1 Literature search

**Figure 1:**
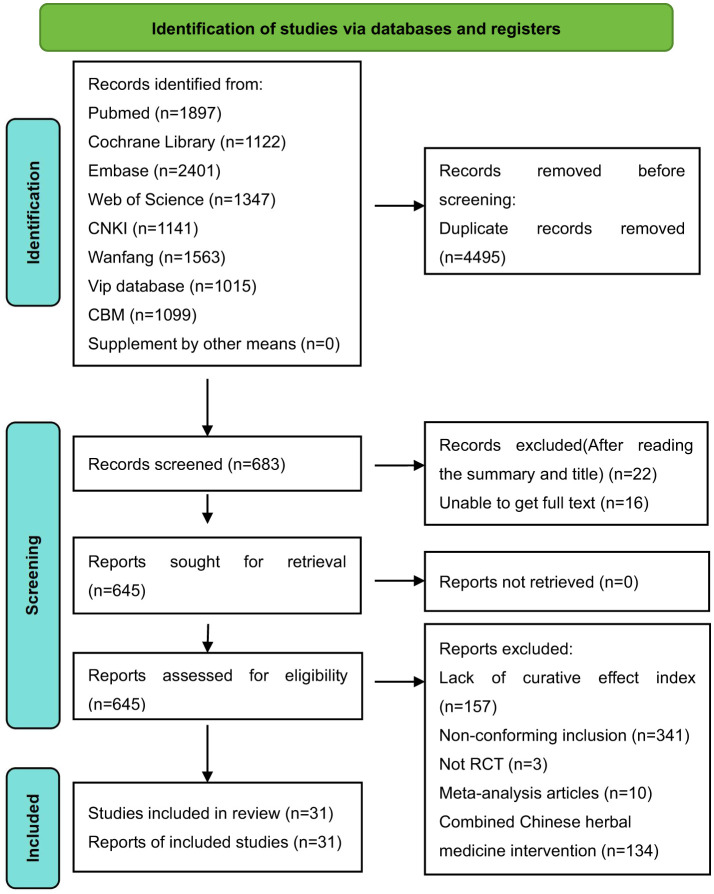
Document screening process and results.

The initial search was conducted in PubMed (*n* = 84), Embase (*n* = 175), Web of Science (*n* = 56), Cochrane Library (*n* = 62), China National Knowledge Infrastructure (CNKI, *n* = 1,141), Wanfang Data (*n* = 1,563), VIP Database (*n* = 1,015), and China Biomedical Literature Database (*n* = 1,099), yielding 5,178 relevant publications. Following multiple rounds of rigorous screening, 31 studies were ultimately included in this meta-analysis: 0 English-language publications and 31 Chinese-language publications, spanning publication years from 2010 to 2025. The literature search and screening process is detailed in Figure 1.

### 3.2 Study characteristics

**Figure 2:**
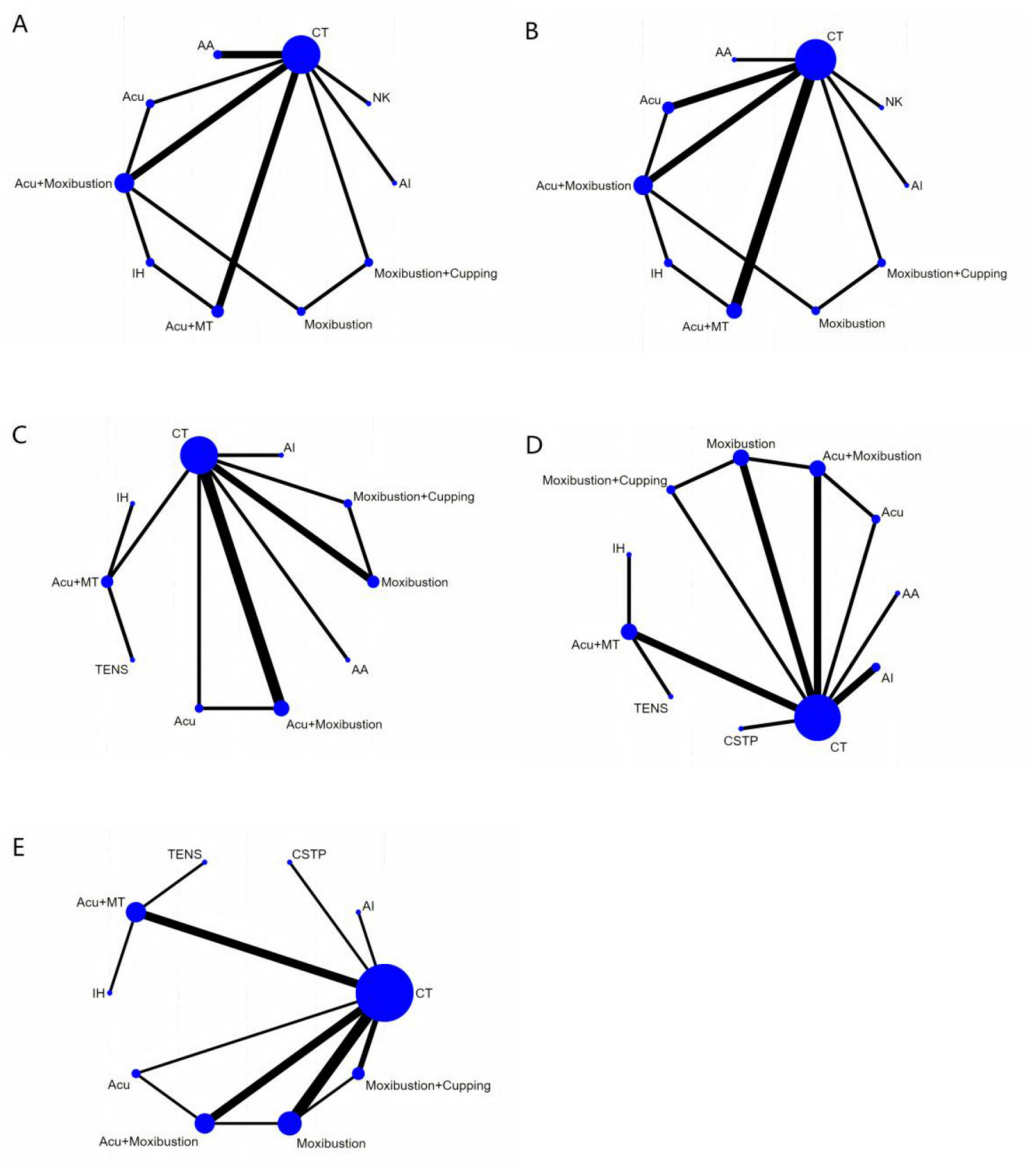
The evidence network of all papers on different treatments. **(A)** BASDAI score; **(B)** BASFI score; **(C)** VAS score; **(D)** CRP score; **(E)** ESR score. The thickness of the lines represents the number of studies, and the sizes of the nodes indicate the total sample sizes for each treatment.

This meta-analysis included 31 studies (14–44), all of which were randomized controlled trials (RCTs). The studies encompassed 15 distinct treatment modalities: conventional therapy (CT), acupoint application (AA), acupuncture (Acu), moxibustion (Moxibustion), acupoint injection (AI), massage therapy (MT), cupping (Cupping), non-interventional therapy (NI), needle-knife therapy (NK), core stability training programme (CSTP), intradermal injection (IH), transcutaneous electrical nerve stimulation (TENS), acupuncture + moxibustion (Acu + Moxibustion), moxibustion + cupping (Moxibustion + Cupping), and acupuncture + massage (Acu + MT). Detailed characteristics of the included studies are presented in Appendix 3. Figure 2 displays the comprehensive network diagram for all outcome measures across different comparisons.

### 3.3 Assessment of study quality

**Figure 3:**
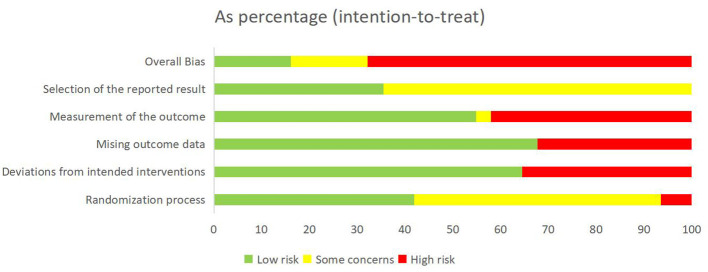
Summary results on the risk of bias of the included RCTs. Percent of studies with categories for risk of bias.

The quality of all 31 randomized controlled trials (RCTs) included in this analysis underwent comprehensive evaluation. Among these, one study employing randomization methods based on registration order, consultation sequence, or outpatient/inpatient numbering was deemed at high risk of potential selection bias (21). In contrast, 15 studies employing random number tables or computer randomization generators for subject allocation were judged to be at low risk (18, 20, 22, 25, 27, 29, 31, 32, 34, 36, 38–41, 43). However, 16 studies, whilst mentioning randomization, lacked detailed information on the randomization process and were therefore categorized as having an unclear risk (14–17, 19, 21, 23, 24, 26, 28, 30, 33, 35, 37, 42, 44). Regarding randomization, although all included studies claimed to be randomized controlled trials, detailed descriptions of sequence generation were frequently lacking. Sixteen studies did not provide sufficient information regarding the randomization procedure and were therefore judged as having unclear risk of bias. Regarding allocation concealment, only two studies adequately described concealment methods, whereas the remaining studies either failed to report relevant procedures or provided insufficient details, resulting in concerns regarding selection bias. In addition, due to the characteristics of non-pharmacological CAM interventions, blinding of participants and personnel was generally difficult to implement, and inadequate reporting of blinding procedures contributed to potential performance and detection bias. Detailed findings are presented in Figure 3.

### 3.4 Meta-analysis

The network geometry is presented in Figure 2. The evidence network consisted of 15 CAM interventions and conventional treatment comparisons. The distribution of available evidence varied across outcomes, with some interventions supported by multiple direct comparisons, whereas others relied mainly on indirect evidence. Conventional treatment was the most frequently connected comparator within the network, while several CAM interventions were evaluated in relatively few studies, resulting in limited evidence connectivity.

#### 3.4.1 BASDAI

**Figure 4:**
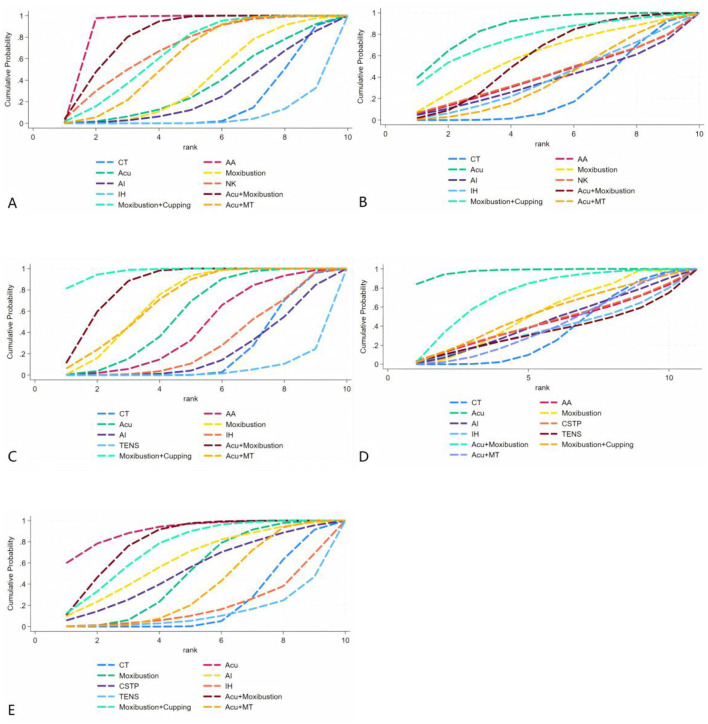
Ranking of the cumulative probabilities for core parameters. **(A)** BASDAI score; **(B)** BASFI score; **(C)** VAS score; **(D)** CRP score; **(E)** ESR score.

Fifteen studies involving 1,257 patients were included in the BASDAI score assessment. The NMA revealed that, among the 10 complementary and alternative medicine (CAM) therapies included, several interventions demonstrated statistically significant improvements in BASDAI scores compared with conventional treatment (CT), including Moxibustion+Cupping (MD = −1.05, 95% CI [−2.07, −0.03]), Acu+MT (MD = −1.14, 95% CI [−2.03, −0.25]), Moxibustion (MD = −1.51, 95% CI [−2.58, −0.44]), Acu (MD = −1.63, 95% CI [−2.85, −0.41]), and AI (MD = −1.81, 95% CI [−2.90, −0.73]). Furthermore, the network comparison indicated significant differences among some interventions. Specifically, Acu+Moxibustion was associated with greater reductions in BASDAI scores compared with NK, Moxibustion+Cupping, Acu+MT, Moxibustion, Acu, AI, CT, and IH. According to the SUCRA analysis, Acu+Moxibustion and NK showed relatively higher probabilities of being among the most effective interventions for improving BASDAI scores. However, these rankings should be interpreted with caution, as SUCRA values represent relative ranking probabilities rather than definitive evidence of treatment superiority. The interpretation of treatment rankings may be affected by factors such as the number of included studies, sample size, network structure, and certainty of evidence. Detailed findings are presented in Table 2 and Figure 4A.

**Table 2:** The SUCRA values of each treatment modality.

Treatment	BASDAI	Rank	BASFI	Rank	VAS	Rank	CRP	Rank	ESR	Rank
CT	17.3%	8	25.0%	10	21.9%	8	34.3%	10	20.7%	8
AA	17.0%	9	41.3%	5	44.0%	6	41.6%	7	–	–
Acu	36.0%	6	86.1%	1	57.0%	5	97.6%	1	90.3%	1
Moxibustion	40.1%	5	60.1%	3	69.9%	4	51.2%	4	50.1%	6
AI	26.9%	7	35.6%	9	21.1%	9	45.4%	5	62.8%	4
MT	–	–	–	–	–	–	–	–	–	–
Cupping	–	–	–	–	–	–	–	–	–	–
NI	–	–	–	–	–	–	–	–	–	–
NK	69.5%	2	41.2%	6	–	–	–	–	–	–
CSTP	–	–	–	–	–	–	44.3%	6	52.4%	5
IH	5.7%	10	37.2%	8	29.4%	7	36.4%	9	18.9%	9
TENS	–	–	–	–	4.7%	10	34.4%	11	12.3%	10
Acu+Moxibustion	79.9%	1	58.5%	4	83.9%	2	73.5%	2	80.0%	2
Moxibustion + Cupping	65.6%	3	77.0%	2	97.2%	1	52.2%	3	74.5%	3

#### 3.4.2 BASFI

In the BASFI assessment, 16 studies involving 1,257 patients were included. The NMA indicated that, among the 10 complementary and alternative medicine (CAM) therapies included, no CAM intervention demonstrated statistically significant superiority over conventional treatment (CT), as all 95% confidence intervals crossed the line of no effect. Although some interventions showed favorable trends in BASFI improvement, the differences compared with CT did not reach statistical significance. Although no CAM intervention showed statistically significant superiority over CT, the SUCRA analysis suggested that Acu (86.1%) and Moxibustion + Cupping (77.0%) had relatively higher probabilities of achieving better BASFI outcomes. This apparent discrepancy may be explained by the fact that SUCRA provides a relative ranking of interventions based on the entire network of evidence, rather than direct evidence of statistically significant differences between specific treatments. Therefore, higher SUCRA probabilities should be interpreted as an indication of potential comparative advantages rather than definitive treatment superiority. The clinical relevance of these findings should be further evaluated by considering effect estimates, confidence intervals, certainty of evidence, and predefined minimal clinically important difference (MCID) thresholds. Detailed findings are shown in Table 2 and Figure 4B.

#### 3.4.3 VAS

Fourteen studies involving 1,118 patients were included in the VAS score assessment. The NMA revealed that, among the 10 complementary and alternative medicine (CAM) therapies included, Moxibustion (MD = −0.76, 95% CI [−1.46, −0.06]), Acu (MD = −1.05, 95% CI [−2.03, −0.07]), AA (MD = −1.37, 95% CI [−2.46, −0.28]), and IH (MD = −1.75, 95% CI [−2.98, −0.52]) demonstrated statistically significant reductions in VAS scores compared with conventional treatment (CT). Furthermore, significant differences were observed among some interventions within the treatment network. Specifically, Acu+Moxibustion was associated with greater reductions in VAS scores compared with Moxibustion, Acu, AA, IH, AI, TENS, and Acu+MT. According to the SUCRA analysis, Moxibustion + Cupping (97.2%) and Acu + Moxibustion (83.9%) showed relatively higher probabilities of achieving favorable outcomes for pain reduction. Although Moxibustion + Cupping demonstrated a statistically significant improvement in pain scores compared with CT, the clinical relevance of this effect should be interpreted according to whether the observed difference exceeds the predefined minimal clinically important difference (MCID) threshold for VAS. Moreover, SUCRA values represent relative ranking probabilities rather than definitive evidence of treatment superiority. Therefore, treatment selection should consider the magnitude of treatment effects, confidence intervals, clinical relevance, and certainty of evidence, rather than relying solely on ranking probabilities. Detailed findings are shown in Table 2 and Figure 4C.

#### 3.4.4 CRP

For CRP assessment, 17 studies involving 1,484 patients were included. The NMA revealed that, among the 10 complementary and alternative medicine (CAM) therapies included, Moxibustion (MD = −40.69, 95% CI [−76.83, −4.55]), AI (MD = −44.34, 95% CI [−85.94, −2.74]), and Acu+MT (MD = −46.91, 95% CI [−86.96, −6.87]) demonstrated statistically significant reductions in CRP levels compared with conventional treatment (CT). Furthermore, significant differences were observed among some interventions within the treatment network. Specifically, Acu+Moxibustion was associated with greater reductions in CRP levels compared with Moxibustion+Cupping, Moxibustion, AI, CSTP, AA, Acu+MT, IH, CT, and TENS. According to the SUCRA rankings, Acu (97.6%) and Acu+Moxibustion (73.5%) showed relatively higher probabilities of being among the most effective interventions for reducing CRP levels. However, SUCRA values represent relative ranking probabilities rather than definitive evidence of treatment superiority. Therefore, these rankings should be interpreted cautiously and considered together with effect estimates, confidence intervals, clinical relevance, and certainty of evidence. Detailed findings are shown in Table 2 and Figure 4D.

#### 3.4.5 ESR

Twenty studies involving 1,642 patients were included in the ESR assessment. The network meta-analysis revealed that, among the 10 complementary and alternative medicine (CAM) therapies included, no CAM intervention demonstrated statistically significant superiority over conventional treatment (CT), as all 95% confidence intervals crossed the line of no effect. However, significant differences were observed among some interventions within the treatment network. Specifically, Acu+Moxibustion was associated with greater reductions in ESR levels compared with Moxibustion+Cupping, AI, CSTP, Acu+MT, Moxibustion, CT, IH, and TENS. Although none of the CAM interventions showed statistically significant superiority over CT in direct comparative analyses, the SUCRA analysis provided additional information regarding the relative ranking of interventions within the entire evidence network. According to the SUCRA rankings, Acu (90.3%) and Acu+Moxibustion (80.0%) showed relatively higher probabilities of achieving favorable outcomes in reducing ESR levels. These higher ranking probabilities indicate a greater likelihood of being among the better-performing interventions, but they do not represent definitive evidence of statistically significant treatment superiority. Therefore, SUCRA-based rankings should be interpreted cautiously and considered together with effect estimates, confidence intervals, clinical relevance, and certainty of evidence. Detailed findings are shown in Table 2 and Figure 4E.

### 3.5 Subgroup analysis

#### 3.5.1 VAS

The forest plot results indicate that two studies were included in the analysis when comparing the clinical efficacy of moxibustion vs. sulfasalazine. Using MD as the effect measure, the estimated overall combined effect size was MD = −1.17 (95% CI: −1.61, −0.74). Since this 95% confidence interval lies entirely to the left of the null line and does not include zero, it demonstrates that the improvement in the moxibustion group was significantly greater than that in the sulfasalazine group at the corresponding clinical outcome endpoint, with a highly statistically significant difference between the two groups.

**Figure 5:**
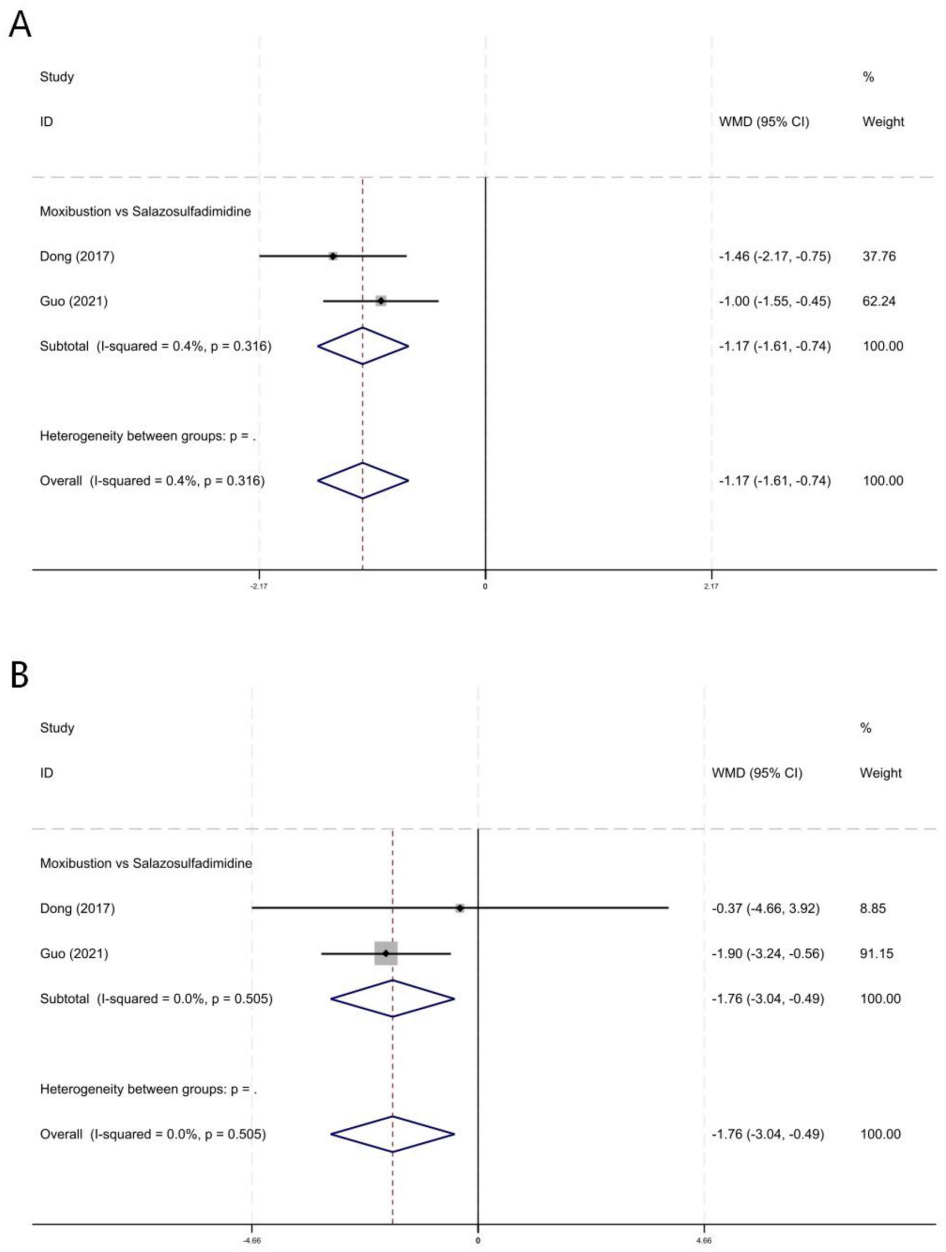
Forest map. **(A)** VAS; **(B)** ESR.

Heterogeneity testing revealed no significant statistical heterogeneity between the two included studies (*I*^2^ = 0.4%, *P* = 0.316), indicating high consistency in both the direction and magnitude of effects across independent studies, thereby demonstrating the strong robustness of the pooled results. In terms of weight allocation, Guo et al.'s (63) study contributed the dominant weight. Based on this evidence, the meta-analysis supports that moxibustion exhibits a more pronounced therapeutic advantage over sulfasalazine in improving the relevant clinical outcomes. Detailed findings are shown in Figure 5A.

#### 3.5.2 ESR

The forest plot results indicate that two studies were included in the analysis when comparing the clinical efficacy of moxibustion vs. sulfasalazine. Using MD as the effect measure, the estimated overall combined effect size was MD = −1.76 (95% CI: −3.04, −0.49). Since the 95% confidence interval for this combined effect size lies entirely to the left of the null line and does not include zero, it demonstrates that the improvement in clinical outcomes was significantly greater in the moxibustion group than in the sulfasalazine group, with a statistically significant difference between the two groups.

Heterogeneity testing revealed no significant statistical heterogeneity between the two included studies (*I*^2^ = 0.0%, *P* = 0.505), indicating high consistency and reproducibility across the independent studies. In terms of weight allocation, Guo's (2021) study contributed the highest absolute weight (91.15%). Based on these findings, this meta-analysis demonstrates that moxibustion exhibits a more pronounced therapeutic advantage over sulfasalazine in improving the relevant clinical outcomes. Detailed findings are shown in Figure 5B.

#### 3.5.3 Summary

In conclusion, this meta-analysis suggests that moxibustion therapy may provide potential benefits compared with sulfasalazine in improving relevant clinical indicators. Although statistical heterogeneity was low, the limited number of included studies (only two), together with the substantial contribution of one study (Guo, 2021) to certain outcomes, reduces confidence in the robustness of the findings. Only predefined subgroup analyses supported by sufficient comparable data were conducted, while other clinically relevant subgroup factors and meta-regression analyses could not be reliably performed because of limited study numbers and inconsistent reporting across trials. Future large-scale, high-quality randomized controlled trials are required to further validate the clinical efficacy and establish the certainty of evidence regarding moxibustion therapy.

### 3.6 Consistency assessment

Consistency was assessed through comparison of direct and indirect evidence, where applicable. No important inconsistency was identified based on available closed loops and consistency evaluation methods. However, the limited number of studies for some comparisons restricted the ability to fully assess inconsistency.

### 3.7 Small-sample assessment

To evaluate whether this network meta-analysis exhibits small-sample effects or publication bias, comparative correction funnel plots were applied to five outcome measures. Overall, the funnel plots for BASDAI, BASFI, and VAS scores indicated that data points representing individual studies were broadly symmetrically distributed on either side of the central vertical line (i.e., the line of the pooled effect size). This symmetry, particularly the near-funnel shape formed by points from studies with higher precision (smaller standard errors), suggests a low likelihood of significant publication bias for these patient-reported outcome measures.

By contrast, the symmetry of funnel plots for the laboratory inflammation markers CRP and ESR was comparatively poorer. The CRP funnel plot exhibited mild asymmetry, with some data points showing a tendency to cluster toward one side of the graph. The ESR funnel plot exhibited more pronounced dispersion, with a few data points featuring large standard errors (typically corresponding to small sample sizes or high heterogeneity) falling outside the expected symmetrical region. This asymmetric pattern suggests that publication bias or small-sample effects cannot be entirely ruled out in analyses of these two outcome measures.

Potential causes include, firstly, the small-sample effect: some included studies had limited sample sizes, leading to potentially greater variability in effect estimates, and negative results from small-sample studies may have gone unpublished. Secondly, measurement and reporting heterogeneity: although CRP and ESR are objective indicators, differences in detection methods, timing of testing, and reference ranges across studies may introduce additional variability. Thirdly, language and retrieval limitations exist. This study included only Chinese and English literature, with ultimately only Chinese-language studies being incorporated. Given that all included studies originated from China and were published in Chinese, the possibility of regional and language-related publication bias cannot be completely excluded, even though funnel plot analyses did not demonstrate substantial asymmetry across most outcomes. This may have omitted relevant research published in other languages, particularly unpublished negative results.

**Figure 6:**
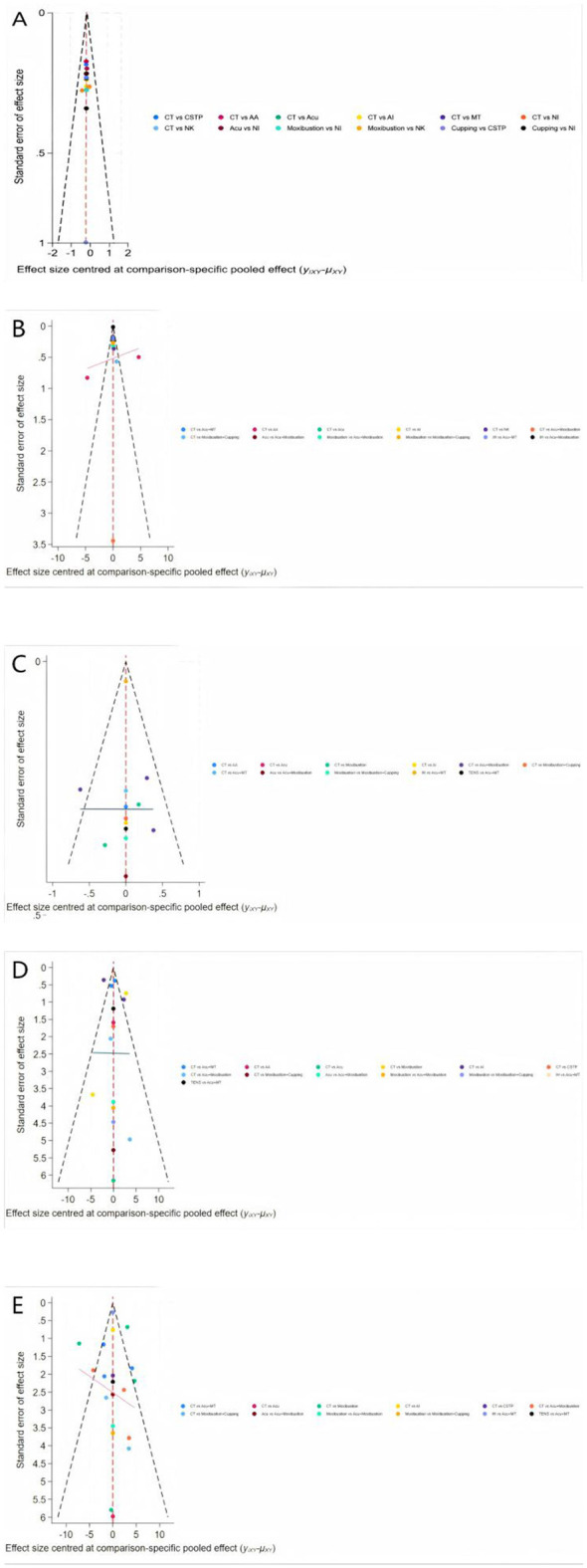
Funnel plot. **(A)** BASDAI score; **(B)** BASFI score; **(C)** VAS score; **(D)** CRP score; **(E)** ESR score.

Nevertheless, the funnel plot patterns across the five outcome measures show most data points clustered around the center line, with no evidence of severe, systematic asymmetry. This lends some support to the overall robustness of the study's primary findings. Interpretation of comparative findings regarding CRP and ESR should be approached with appropriate caution. Future research in this field requires larger-scale, high-quality studies to further validate and consolidate current conclusions regarding improvements in inflammatory markers. Observed results are presented in Figure 6.

### 3.8 CINeMA

We employed the Network Meta-Analytic Confidence Interval (CINeMA) method to evaluate all paired comparisons related to the three outcome measures. For the BASDAI score, the evidence quality supporting the efficacy of IH: Acupuncture + Moxibustion, IH: Acupuncture + Moxibustion, AA: NK, IH: AA, Acupuncture: NK, Acupuncture + MT: Moxibustion, and Acupuncture + Moxibustion: NK in reducing the BASDAI score was assessed as “moderate,” indicating a reasonable level of confidence regarding the therapeutic benefits of these interventions. Regarding the BASFI score, the evidence quality for the efficacy of CT: AI, CT: Acupuncture + Moxibustion, Moxibustion + Cupping: Moxibustion, CT: Moxibustion, AA: Moxibustion, Acupuncture: Moxibustion, IH: Moxibustion, AI: NK, Moxibustion + Cupping: AI, IH: NK, and IH: Moxibustion + Cupping in reducing the BASFI score was also rated as “moderate.” Similarly, regarding VAS scores, the quality of evidence for the effects of CT: Moxibustion + Cupping; TENS: Acupuncture + Moxibustion; Acupuncture + Moxibustion: Acupuncture alone; AA: Moxibustion; AA: Acupuncture alone; Acupuncture: Moxibustion; Acupuncture + Moxibustion: Acupuncture alone; Acupuncture + Moxibustion: Moxibustion; IH: Acupuncture alone; TENS: Acupuncture + Moxibustion; and Acupuncture + Moxibustion: Acupuncture + Moxibustion were all assessed as “moderate.” The evidence quality for these therapies is relatively robust. In contrast, the comparative evidence quality among other therapies was rated as “low” or “very low,” reflecting significant uncertainty. These conclusions should be applied with caution in clinical practice. Further detailed evaluation results are presented in Supplementary Tables S6.1–S6.3. Based on these findings, we deem it necessary to conduct additional high-quality studies to further confirm the efficacy of complementary and alternative therapies for patients with AS. Importantly, some comparisons demonstrated statistically significant differences but did not necessarily exceed the predefined MCID thresholds, indicating that statistical evidence alone may not represent meaningful clinical benefit. Therefore, treatment effects were interpreted by considering both statistical uncertainty and clinical relevance.

## 4 Discussion

### 4.1 Summary of key findings

This network meta-analysis included 31 randomized controlled trials involving 2,459 patients with ankylosing spondylitis and compared 15 CAM interventions. Compared with conventional treatment, several CAM therapies demonstrated statistically favorable effects across disease activity, pain, functional status, and inflammatory outcomes. However, the clinical relevance of these effects varied and should be interpreted according to MCID thresholds and the certainty of evidence. However, these findings should be interpreted in the context of clinical heterogeneity among included trials, including variations in control conditions, treatment regimens, treatment duration, and concomitant medication use.

Based on the clinical outcomes evaluated in this network meta-analysis, several CAM interventions showed potential benefits for symptom improvement and inflammatory control. Existing mechanistic studies may provide possible biological explanations for these findings; however, such mechanisms require further validation.

For disease activity (BASDAI), acupoint injection, acupuncture, moxibustion, acupuncture plus massage, and moxibustion plus cupping showed greater reductions than conventional treatment, with mean differences ranging from −1.05 to −1.81 points. Given that a reduction of approximately 1 unit in BASDAI is generally considered clinically meaningful, these findings suggest that several CAM interventions may be associated with symptom improvement; however, the magnitude and clinical relevance of these effects remain uncertain due to limitations in evidence certainty (45, 46).

For pain outcomes (VAS), intradermal injection, acupoint application, acupuncture, and moxibustion achieved statistically significant reductions compared with conventional therapy, with effect sizes ranging from −0.76 to −1.75 points. These results indicate that CAM interventions may contribute to pain reduction; however, whether these effects represent clinically meaningful improvements depends on comparison with established MCID thresholds.

Regarding inflammatory markers, acupuncture-based interventions showed relatively favorable effect estimates for CRP and ESR outcomes; however, these findings should be interpreted cautiously because evidence certainty was limited. Acupuncture combined with moxibustion and acupuncture alone showed the most favorable effect estimates across inflammatory outcomes, suggesting a potential role in controlling systemic inflammation.

Importantly, although SUCRA rankings suggested that certain interventions had higher probabilities of achieving favorable outcomes, these rankings should not be interpreted as definitive evidence of clinical superiority. SUCRA values provide relative rankings within the available evidence network but do not directly incorporate the certainty of evidence, the clinical importance of effect sizes, or the potential influence of study limitations. Therefore, the interpretation of treatment effects in this study was primarily based on estimated effect sizes, confidence intervals, clinical relevance, and CINeMA/GRADE certainty assessments rather than ranking probabilities alone (47, 48). Therefore, the present findings should primarily be interpreted according to effect estimates, confidence intervals, and clinical relevance rather than SUCRA values alone.

### 4.2 Potential mechanisms underlying CAM effects

The present network meta-analysis identified potential benefits of several CAM interventions for improving disease activity, pain, physical function, and inflammatory markers in patients with ankylosing spondylitis. Although the current study was not designed to investigate biological mechanisms, previous experimental and clinical studies suggest that these effects may involve multiple regulatory pathways (49–51).

Potential mechanisms include modulation of inflammatory responses, regulation of immune cell balance, attenuation of pain sensitization, and improvement of local tissue microcirculation (49–51). For example, acupuncture has been reported to regulate inflammatory cytokine release and neuroimmune interactions, thereby contributing to its analgesic and anti-inflammatory effects (49, 50). Moxibustion may influence inflammatory responses through modulation of cytokine profiles, although the underlying mechanisms remain incompletely understood (51). Cupping therapy may contribute to pain relief through mechanical stimulation and local physiological responses; however, the evidence supporting its biological mechanisms remains preliminary (52). However, these proposed mechanisms remain hypothesis-generating, as the present network meta-analysis evaluated clinical outcomes rather than molecular or cellular biomarkers.

Therefore, mechanistic interpretations should be considered complementary explanations for the observed clinical effects rather than direct evidence derived from this study. Further translational research integrating clinical outcomes with biological measurements is required to clarify the mechanisms underlying CAM interventions in ankylosing spondylitis.

### 4.3 Clinical interpretation and implications

The present findings provide preliminary insights into the potential role of CAM interventions in the management of ankylosing spondylitis; however, their implications for clinical practice should be interpreted cautiously given the limited certainty of evidence.

According to the current ASAS-EULAR recommendations for the management of axial spondyloarthritis, pharmacological treatments, including non-steroidal anti-inflammatory drugs (NSAIDs) and biologic or targeted synthetic disease-modifying therapies for eligible patients, remain the cornerstone of disease management, while non-pharmacological interventions such as exercise and patient education are also recommended as important components of comprehensive care. CAM interventions are not currently recommended as substitutes for established pharmacological therapies but may be considered as potential complementary approaches for symptom management in selected patients. Therefore, the findings of this network meta-analysis should be interpreted within the framework of guideline-based care, suggesting that CAM therapies may provide additional supportive benefits rather than replace evidence-based standard treatments.

First, the results suggest that CAM interventions may serve as potential adjunctive approaches within multidisciplinary management of ankylosing spondylitis. However, given the limited certainty of evidence, they should not currently be considered substitutes for established pharmacological or guideline-recommended therapies (53). Several interventions demonstrated benefits across multiple clinically important outcomes, including disease activity, pain, physical function, and inflammatory biomarkers.

Second, different interventions appeared to exhibit distinct therapeutic profiles (54). Acupoint application and acupoint injection showed relatively greater effects on disease activity; acupuncture demonstrated more consistent improvements in inflammatory markers and functional outcomes, whereas moxibustion combined with cupping showed favorable effects on pain relief. These differences may provide preliminary insights into individualized symptom management; however, differences in statistical estimates should not be interpreted as definitive evidence of clinically important superiority.

Third, from a patient-centered perspective, some CAM therapies are relatively accessible and may be acceptable to patients because of their non-pharmacological characteristics (55). However, conclusions regarding their safety should be interpreted cautiously because adverse event reporting was limited and inconsistent across the included studies. The absence of reported serious adverse events should not be interpreted as evidence of confirmed safety. Therefore, although CAM interventions may represent potential supplementary options for selected patients, their safety requires further evaluation through well-designed clinical trials with standardized adverse event reporting.

Following the GRADE Working Group recommendations, our findings can be summarized using informative statements rather than treatment rankings alone. The available evidence suggests that acupuncture-based interventions may reduce inflammatory activity and improve physical function, while moxibustion combined with cupping may reduce pain intensity. However, the certainty of evidence remains limited because most included studies were small single-center trials with methodological limitations. Consequently, these interventions should currently be considered promising adjunctive options rather than definitive replacements for guideline-recommended pharmacological treatment (56, 57).

### 4.4 Limitations

Several limitations should be considered when interpreting the findings of this network meta-analysis. First, all included studies were conducted in China and published in Chinese, which may limit the generalizability of the results to other populations with different healthcare systems, ethnic backgrounds, and clinical practice settings. Although comprehensive searches were performed across multiple databases, the available evidence may still be influenced by geographic and language-related publication bias.

Second, the overall certainty of evidence was predominantly low or very low according to the CINeMA/GRADE assessment. This was mainly attributable to methodological limitations of the included randomized controlled trials, including insufficient reporting of random sequence generation, allocation concealment, and blinding procedures. Although blinding is difficult to implement for acupuncture and related interventions, future clinical trials should adopt more rigorous methodological designs and follow standardized reporting recommendations, such as the CONSORT guidelines, to improve the transparency and reliability of evidence generation (58).

Third, the interpretation of network meta-analysis findings requires caution. Although network geometry and consistency assessments were performed according to recommended methodological approaches, the evidence network for some interventions was relatively sparse. Differences in CAM protocols, treatment duration, concomitant medications, and baseline disease characteristics may have influenced the transitivity assumption and contributed to residual heterogeneity. Therefore, indirect comparisons and treatment rankings should be interpreted cautiously and require further confirmation through well-designed head-to-head randomized controlled trials (59).

Fourth, the clinical interpretation of some outcomes remains limited. Although BASDAI, BASFI, and pain VAS are widely used and clinically meaningful outcome measures in ankylosing spondylitis, inflammatory biomarkers such as CRP and ESR lack universally accepted minimal clinically important difference thresholds, limiting the assessment of their clinical relevance. In addition, adverse events were insufficiently reported in many included studies, preventing a comprehensive evaluation of the safety profile of CAM interventions. Future studies should incorporate standardized safety monitoring and reporting procedures.

### 4.5 Clinical implications

Despite these limitations, the findings of this network meta-analysis provide preliminary evidence that several CAM interventions may offer potential benefits for patients with ankylosing spondylitis. Current treatment recommendations emphasize the importance of individualized management strategies combining pharmacological and non-pharmacological approaches, with conventional therapies remaining the cornerstone of disease control (53, 60). CAM interventions may therefore serve as complementary approaches for symptom management and functional improvement rather than replacements for established treatments.

The present findings suggest that different CAM modalities may provide potential benefits for specific clinical outcomes. For example, acupuncture-based interventions have demonstrated potential effects in chronic pain management, supporting their possible role in alleviating pain-related symptoms in patients with musculoskeletal disorders (55, 61). However, the observed treatment effects should be interpreted together with the certainty of evidence, magnitude of effect estimates, and clinical relevance.

Future research should focus on large-scale, multicenter randomized controlled trials with standardized intervention protocols, longer follow-up periods, improved methodological quality, and comprehensive safety assessment. In addition, future studies should explore whether specific patient characteristics, disease stages, or treatment combinations influence responses to CAM interventions, thereby improving the precision and clinical applicability of individualized treatment strategies (62).

## 5 Conclusions

This network meta-analysis synthesized comparative evidence from 31 randomized controlled trials involving 2,459 patients with ankylosing spondylitis and evaluated 15 complementary and alternative medicine (CAM) interventions. The findings suggest that several CAM therapies may provide beneficial effects on disease activity, pain, functional status, and inflammatory outcomes. Different CAM modalities showed potential advantages across specific clinical outcomes, providing valuable evidence for the optimization of individualized treatment strategies.

The results indicate that CAM interventions may serve as complementary approaches within the comprehensive management of ankylosing spondylitis, particularly for symptom relief and functional improvement. The interpretation of treatment effects should consider both statistical significance and clinical relevance. Future high-quality, multicenter randomized controlled trials with standardized intervention protocols and long-term follow-up are warranted to further validate the effectiveness and safety of CAM therapies.

## Data Availability

The original contributions presented in the study are included in the article/Supplementary material, further inquiries can be directed to the corresponding author.
