# Supplementary material for: The efficacy of complementary and alternative medicine non-pharmacological interventions in improving disease activity and inflammation in ankylosing spondylitis: a network meta-analysis
Source: Front Med (Lausanne). 2026 Aug 3;13:1824982. doi: 10.3389/fmed.2026.1824982 (PMC13477885; doi:10.3389/fmed.2026.1824982)
Supplement: Supplementary file 1 [file Supplementary_file_1.docx]

Supplementary Appendix

**The Efficacy of Complementary and Alternative Medicine Non-Pharmacological Interventions in Improving Disease Activity and Inflammation in Ankylosing Spondylitis: A Network Meta-Analysis**

**Table of contents**

**Appendix 1. Search Strategy	2**

**Appendix 2. Calculation Formulas for Numerical Data Conversion	14**

**Appendix 3. Characteristics of Included Studies	15**

**Appendix 4. Risk of Bias of Randomized Clinical Trials	23**

**Appendix 5. League Table	24**

**Appendix 6. GRADE	34**

**Appendix 7. RoB	46**

**Appendix 1. Search Strategy**

**Table S1.1.** Search Strategy for PubMed：

#1 Ankylosing Spondylitis

("Spondylitis, Ankylosing"[Mesh]

OR "ankylosing spondylitis"[tiab]

OR "ankylosing spondyloarthritis"[tiab]

OR "ankylosing spondylarthritis"[tiab]

OR "bechterew disease"[tiab]

OR "bechterew's disease"[tiab]

OR "marie struempell disease"[tiab]

OR "marie-struempell disease"[tiab]

OR "rheumatoid spondylitis"[tiab]

OR "spondylitis ankylopoietica"[tiab]

OR "spondylarthritis ankylopoietica"[tiab]

OR "spondyloarthritis ankylopoietica"[tiab])

#2 Chinese Medicine Non-pharmacological Interventions

("Acupuncture Therapy"[Mesh]

OR "Moxibustion"[Mesh]

OR "Massage"[Mesh]

OR acupuncture[tiab]

OR electroacupuncture[tiab]

OR electro-acupuncture[tiab]

OR abdominal acupuncture[tiab]

OR umbilical acupuncture[tiab]

OR navel acupuncture[tiab]

OR auricular acupuncture[tiab]

OR auricular needling[tiab]

OR filiform needle[tiab]

OR acupoint[tiab]

OR acupoint stimulation[tiab]

OR tuina[tiab]

OR massage[tiab]

OR moxibustion[tiab]

OR cupping[tiab]

OR cupping therapy[tiab]

OR acupotomy[tiab]

OR needle knife[tiab])

#3 Randomized Controlled Trials

("Randomized Controlled Trial"[Publication Type]

OR "Controlled Clinical Trial"[Publication Type]

OR randomized[tiab]

OR randomised[tiab]

OR randomly[tiab]

OR random allocation[tiab]

OR placebo[tiab]

OR sham[tiab]

OR trial[tiab]

OR clinical trial[tiab]

OR controlled trial[tiab]

OR assessor blind*[tiab]

OR single blind*[tiab]

OR double blind*[tiab])

#4

#1 AND #2 AND #3

**Table S1.2.** Search Strategy for the Cochrane Library

#1 Ankylosing Spondylitis

(Spondylitis, Ankylosing)

OR (Ankylosing Spondylitis)

OR (Ankylosing Spondyloarthritis)

OR (Ankylosing Spondylarthritis)

OR (Bechterew Disease)

OR (Bechterew's Disease)

OR (Marie-Struempell Disease)

OR (Marie Struempell Disease)

OR (Rheumatoid Spondylitis)

OR (Spondylitis Ankylopoietica)

OR (Spondylarthritis Ankylopoietica)

OR (Spondyloarthritis Ankylopoietica)

OR (Spondylitis, Rheumatoid)

#2 Traditional Chinese Medicine Non-pharmacological Interventions

(Electroacupuncture)

OR (Electro-acupuncture)

OR (Acupuncture)

OR (Abdominal Acupuncture)

OR (Umbilical Acupuncture)

OR (Navel Acupuncture)

OR (Auricular Acupuncture)

OR (Auricular Needling)

OR (Filiform Needle)

OR (Acupoint)

OR (Acupoint Stimulation)

OR (Massage)

OR (Tuina)

OR (Moxibustion)

OR (Cupping)

OR (Cupping Therapy)

OR (Acupotomy)

OR (Needle Knife)

#3 Randomized Controlled Trials

(Randomized Controlled Trial)

OR (Randomised Controlled Trial)

OR (Controlled Clinical Trial)

OR (Clinical Trial)

OR (Controlled Trial)

OR (Randomized)

OR (Randomised)

OR (Randomly)

OR (Random Allocation)

OR (Placebo)

OR (Sham)

OR (RCT)

#4

#1 AND #2 AND #3

**Table S1.3.** Search Strategy for Embase

#1 Ankylosing Spondylitis

('ankylosing spondylitis'/exp

OR 'bechterew disease'/exp

OR 'ankylosing spondylitis':ti,ab,kw

OR 'bechterew disease':ti,ab,kw

OR 'marie struempell disease':ti,ab,kw

OR 'rheumatoid spondylitis':ti,ab,kw

OR 'ankylosing spondyloarthritis':ti,ab,kw)

#2 CAM Interventions

('acupuncture'/exp

OR 'electroacupuncture'/exp

OR 'moxibustion'/exp

OR 'massage'/exp

OR 'tuina'/exp

OR 'cupping therapy'/exp

OR 'acupotomy'/exp

OR acupuncture:ti,ab,kw

OR electroacupuncture:ti,ab,kw

OR abdominal acupuncture:ti,ab,kw

OR auricular acupuncture:ti,ab,kw

OR moxibustion:ti,ab,kw

OR tuina:ti,ab,kw

OR cupping:ti,ab,kw

OR acupotomy:ti,ab,kw

OR needle knife:ti,ab,kw)

#3 RCT

('randomized controlled trial'/exp

OR 'controlled clinical trial'/exp

OR random*:ti,ab,kw

OR placebo*:ti,ab,kw

OR sham*:ti,ab,kw

OR trial*:ti,ab,kw)

#4

#1 AND #2 AND #3

**Table S1.4.** Search Strategy for Web of Science

#1 TS=(

"ankylosing spondylitis"

OR "ankylosing spondyloarthritis"

OR "bechterew* disease"

OR "marie-struempell disease"

OR "rheumatoid spondylitis"

)

#2 TS=(

acupuncture

OR electroacupuncture

OR "electro-acupuncture"

OR "abdominal acupuncture"

OR "auricular acupuncture"

OR moxibustion

OR tuina

OR massage

OR cupping

OR acupotomy

OR "needle knife"

)

#3 TS=(

randomized

OR randomised

OR random*

OR placebo

OR sham

OR trial

OR "controlled trial"

OR "clinical trial"

OR RCT

)

#4

#1 AND #2 AND #3

**Table S1.5.** Search Strategy for CNKI

( (TI='强直性脊柱炎' OR KY='强直性脊柱炎' OR SU='强直性脊柱炎' OR TI='类风湿性脊柱炎' OR SU='类风湿性脊柱炎' OR TI='别赫捷列夫病' OR TI='Marie-Struempell病') AND (TI='电针' OR KY='电针' OR SU='电针' OR TI='腹针' OR KY='腹针' OR TI='脐针' OR KY='脐针' OR TI='耳针' OR KY='耳针' OR TI='毫针' OR KY='毫针' OR TI='针刺' OR KY='针刺' OR SU='针刺' OR TI='针灸' OR KY='针灸' OR SU='针灸' OR TI='穴位' OR KY='穴位' OR SU='穴位' OR TI='按摩' OR KY='按摩' OR TI='推拿' OR KY='推拿' OR SU='推拿' OR TI='艾灸' OR KY='艾灸' OR SU='艾灸' OR TI='拔罐' OR KY='拔罐' OR SU='拔罐' OR TI='针刀' OR KY='针刀' OR SU='针刀') ) AND ( TI='随机' OR KY='随机' OR SU='随机' OR TI='RCT' OR KY='RCT' OR AB='随机对照试验' OR AB='randomized controlled trial' )

**Table S1.6.** Search Strategy for Wanfang

( (标题:强直性脊柱炎 or 关键词:强直性脊柱炎 or 摘要:强直性脊柱炎) or (标题:类风湿性脊柱炎 or 摘要:类风湿性脊柱炎) ) and ( (标题:电针 or 关键词:电针 or 摘要:电针) or (标题:腹针 or 关键词:腹针) or (标题:脐针 or 关键词:脐针) or (标题:耳针 or 关键词:耳针) or (标题:毫针 or 关键词:毫针) or (标题:针刺 or 关键词:针刺 or 摘要:针刺) or (标题:针灸 or 关键词:针灸 or 摘要:针灸) or (标题:穴位 or 关键词:穴位 or 摘要:穴位) or (标题:按摩 or 关键词:按摩) or (标题:推拿 or 关键词:推拿 or 摘要:推拿) or (标题:艾灸 or 关键词:艾灸 or 摘要:艾灸) or (标题:拔罐 or 关键词:拔罐 or 摘要:拔罐) or (标题:针刀 or 关键词:针刀 or 摘要:针刀) ) and ( 标题:随机 or 关键词:随机 or 摘要:随机 or 标题:RCT or 关键词:RCT or 摘要:随机对照试验 )

**Table S1.7.** Search Strategy for VIP database

((T=强直性脊柱炎 or K=强直性脊柱炎 or R=强直性脊柱炎) or (T=类风湿性脊柱炎 or R=类风湿性脊柱炎) or T=别赫捷列夫病 or T=Marie-Struempell病) and ((T=电针 or K=电针 or R=电针) or (T=腹针 or K=腹针) or (T=脐针 or K=脐针) or (T=耳针 or K=耳针) or (T=毫针 or K=毫针) or (T=针刺 or K=针刺 or R=针刺) or (T=针灸 or K=针灸 or R=针灸) or (T=穴位 or K=穴位 or R=穴位) or (T=按摩 or K=按摩) or (T=推拿 or K=推拿 or R=推拿) or (T=艾灸 or K=艾灸 or R=艾灸) or (T=拔罐 or K=拔罐 or R=拔罐) or (T=针刀 or K=针刀 or R=针刀)) and (T=随机 or K=随机 or R=随机 or T=RCT or K=RCT or R=随机对照试验)

**Table S1.8.** Search Strategy for CBM

((("强直性脊柱炎"[中文标题:智能] OR "强直性脊柱炎"[关键词] OR "强直性脊柱炎"[主题词] OR "类风湿性脊柱炎"[中文标题:智能] OR "类风湿性脊柱炎"[主题词] OR "别赫捷列夫病"[中文标题:智能] OR "Marie-Struempell病"[中文标题:智能]) AND ("电针"[中文标题:智能] OR "电针"[关键词] OR "电针"[主题词] OR "腹针"[中文标题:智能] OR "腹针"[关键词] OR "脐针"[中文标题:智能] OR "脐针"[关键词] OR "耳针"[中文标题:智能] OR "耳针"[关键词] OR "毫针"[中文标题:智能] OR "毫针"[关键词] OR "针刺"[中文标题:智能] OR "针刺"[关键词] OR "针刺"[主题词] OR "针灸"[中文标题:智能] OR "针灸"[关键词] OR "针灸"[主题词] OR "穴位"[中文标题:智能] OR "穴位"[关键词] OR "穴位"[主题词] OR "按摩"[中文标题:智能] OR "按摩"[关键词] OR "推拿"[中文标题:智能] OR "推拿"[关键词] OR "推拿"[主题词] OR "艾灸"[中文标题:智能] OR "艾灸"[关键词] OR "艾灸"[主题词] OR "拔罐"[中文标题:智能] OR "拔罐"[关键词] OR "拔罐"[主题词] OR "针刀"[中文标题:智能] OR "针刀"[关键词] OR "针刀"[主题词]))) AND ("随机对照试验"[文献类型])

**Appendix 2. Calculation Formulas for Numerical Data Conversion**

**Table S2.1.** The sample size conversion formula is required in this study*

| Purpose of using a formula | Formula | Alphabetic interpretation |
| --- | --- | --- |
| Merge Subgroup Data | SD=$\sqrt{\frac{\text{(}\text{N}_{\text{1}}\text{−1)S}\text{D}_{\text{1}}^{\text{2}}\text{+(}\text{N}_{\text{2}}\text{−1)S}\text{D}_{\text{2}}^{\text{2}}\text{+}\frac{\text{N}_{\text{1}}\text{N}_{\text{2}}}{\text{N}_{\text{1}}\text{+}\text{N}_{\text{2}}}\text{(}\text{M}_{\text{1}}^{\text{2}}\text{+}\text{M}_{\text{2}}^{\text{2}}\text{−2}\text{M}_{\text{1}}\text{M}_{\text{2}}\text{)}}{\text{N}_{\text{1}}\text{+}\text{N}_{\text{2}}\text{−1}}}$  N=N₁+N₂  M=(N₁M₁+N₂M₂)/(N₁+N₂) | N1: Sample size of subgroup A  M1: Mean of subgroup A  SD1: Standard deviation of subgroup A  N2: Sample size of subgroup B  M2: Mean of subgroup B  SD2: Standard deviation of subgroup B  SD: Merge standard deviation  N: Merge sample size  M: Merge mean |

*The calculation formulas used in the study refer to the Cochrane Handbook for Systematic Reviews of Interventions. Reference: Higgins JPT, Thomas J, Chandler J, Cumpston M, Li T, Page MJ, Welch VA (editors). Cochrane Handbook for Systematic Reviews of Interventions version 6.4 (updated August 2023). Cochrane, 2023. Available from www.training.cochrane.org/handbook.

**Appendix 3. Characteristics of Included Studies**

**Table S3.1.** Baseline characteristics of included Studies

| Literature | Diagnostic criteria | Sample size (control group/treatment group) | Gender  (Male/Female) | Control group | Treatment group | Intervention measures  (Control group) | Intervention measures  (Treatment group) | Age  (years)  Control group | Age  (years) Treatment group | Treatment duration  (weeks) | Frequency  (times per week) | The End | Randomisation method |
| --- | --- | --- | --- | --- | --- | --- | --- | --- | --- | --- | --- | --- | --- |
| Jin2013 | CMA2010 | 58/52 | 72/38 | 37/21 | 35/17 | CT | AA | 30.53±3.64 | 31.57±3.45 | 24 | 1 | ① | Random number table |
| Yue2014 | ACR1984 | 72/72 | 114/30 | 56/16 | 58/14 | CT | AA | 30.04±6.74 | 28.58±7.32 | 12 | 1 | ①②③④ | Random |
| Huang2016 | ACR1984 | 43/43 | 44/42 | 21/22 | 23/20 | CT | Acu | 47.8±4.2 | 48.0±4.1 | 5 | 2 | ①②④⑤ | Random |
| Ren2020 | Unclear | 30/30 | 40/20 | 19/11 | 21/9 | CT | Acu+Moxibustion | 34.09±5.96 | 32.03±6.17 | 12 | Once every 2 weeks | ①②③ | Random number table |
| Rong2023 | Unclear | 30/30 | 41/19 | 21/9 | 20/10 | CT+Acu+NI | CT+Acu+NI+Moxibustion | 33.13±1.37 | 32.79±1.21 | 4 | 2 | ③④⑤ | Random number table |
| Chen2020 | Unclear | 34/34 | 41/27 | 19/15 | 22/12 | CT | AI | 28.07±7.60 | 31.80±7.85 | 48 | 1 | ①②④ | Random |
| Deng2021 | Unclear | 64/64 | 89/39 | 43/21 | 46/18 | CT | Acu+MT | 33.5±5.3 | 34.4±5.2 | 8 | 1 | ①②③④⑤ | Random number table |
| Dong2017 | Unclear | 30/30 | 48/12 | 23/7 | 25/5 | CT | Moxibustion | 33.62±x | 32.86±x | 12 | Once every 4 weeks | ③④⑤ | Random |
| Gao2025 | Unclear | 30/30 | 39/21 | 19/11 | 20/10 | Moxibustion | Moxibustion+Cupping | 41.15±13.43 | 39.72±13.73 | 2 | 3 | ①②③④⑤ | Random number table |
| Gao2013 | ACR1984 | 60/60 | - | - | - | CT | Acu+Moxibustion | - | - | 4 | 7 | ③④⑤ | Random |
| Guo2021 | ACR1984 | 45/45 | 78/12 | 38/7 | 40/5 | CT | Moxibustion | 30.6±5.2 | 30.9±5.5 | 12 | Once every 4 weeks | ③⑤ | Random |
| He2020 | Unclear | 30/30 | 38/22 | 20/10 | 18/12 | Acu | Acu+Moxibustion | 30.29±5.72 | 30.27±5.70 | 11 | 7 | ③ | Random number table |
| Hu2021 | ACR1984 | 40/40 | 68/12 | 35/5 | 33/7 | CT | CT+AA | 25.6±3.6 | 25.4±3.8 | 2 | 7 | ③ | Random number table |
| Li2023 | Unclear | 20/20 | 37/3 | 18/2 | 19/1 | CT | CT+Moxibustion | 34.47±2.53 | 34.53±2.59 | 12 | 3 | ③ | Random |
| Li2021 | ACR1984 | 40/40 | 62/18 | 30/10 | 32/8 | CT | CT+Cupping+Moxibustion | 34.52±4.17 | 35.76±4.50 | 10 | 1 | ①②③④⑤ | Random number table |
| Li2020 | Unclear | 50/50 | 75/25 | 37/13 | 38/12 | CT | CT+Moxibustion | - | - | 12 | 2 | ④⑤ | Random number table |
| Song2017 | Unclear | 40/40 | 55/25 | 28/12 | 27/13 | CT | Acu+Moxibustion | 32.4±6.9 | 32.8±6.7 | 4 | 3 | ④⑤ | Random |
| Teng2021 | Unclear | 45/45 | 56/34 | 29/16 | 27/18 | Acu | Acu+Moxibustion | 31.3±6.6 | 30.8±6.9 | 3 | 1 | ①②④⑤ | Random number table |
| Wang2022 | Unclear | 45/45 | 62/28 | 32/13 | 30/15 | CT | CT+MT+Acu | 48.6±3.3 | 49.3±3.4 | 4 | 7 | ①②⑤ | Random |
| Wang2016 | Unclear | 46/46 | 58/34 | 28/18 | 30/16 | CT+Acu | CT+Acu+Moxibustion | 31.5±5 | 33±6 | 8 | 6 | ④⑤ | Random number table |
| Wang WY2022 | ACR1984 | 30/30 | 37/23 | 19/11 | 18/12 | CT | CT+Acu+Moxibustion | 38.37±7.53 | 36.13±8.15 | 8 | 1 | ①②③⑤ | Random number table |
| Yan2013 | Unclear | 60/60 | 72/48 | 38/22 | 34/26 | CT | CT+NK | 21.23±1.8 | 20.15±2.0 | 4 | Once every 4 weeks | ④⑤ | Random |
| Yang2015 | Unclear | 40/40 | - | - | - | CT | NK | 34.10±5.19 | 31.90±8.34 | 12 | 1 | ①② | Random number table |
| Zhang2016 | Unclear | 38/38 | 44/32 | 20/18 | 24/14 | CT+CSTP | CT+CSTP+Moxibustion | 37.37±1.05 | 37.84±1.15 | 12 | 6 | ①②③④⑤ | Random number table |
| zhang2021 | ACR1984 | 25/25 | 43/7 | 22/3 | 21/4 | CT | CT+AA | 38.56±11.42 | 39.36±13.59 | 10 | Once every 3 weeks | ② | Random number table |
| Zhou2021 | ACR1984 | 26/26 | 44/8 | 23/3 | 21/5 | CT | CT+Acu | 44.39±8.47 | 43.67±7.80 | 12 | 3 | ⑤ | Random |
| Zuo2018 | ACR1984 | 30/30 | 33/27 | 17/13 | 16/14 | CT | Moxibustion | 28±1 | 27±1 | 4 | 7 | ④⑤ | Random |
| Chen2016 | ACR1984 | 27/28 | 43/12 | 21/6 | 22/6 | CT+Acu | CT+Acu+Moxibustion | 32±7 | 33±6 | 12 | 5 | ①② | Random |
| Li2010 | Unclear | 39/39 | 69/9 | 34/5 | 35/4 | CT | Moxibustion+CSTP | 28.5±4.5 | 26.5±3.1 | 12 | 1 | ⑤ | Random |
| Ma2022 | Unclear | 35/35 | 53/17 | 26/9 | 27/8 | IH | IH+Acu+Moxibustion | 30±9 | 30±98 | 3 | 7 | ①② | Random |
| Xie2015 | ACR1984 | 30/30 | 53/7 | 27/3 | 26/4 | CT | CT+AA+TENS | 27±8 | 25±9 | 8 | 3 | ②④⑤ | Random |

Note: ACR: American College of Rheumatology; CMA:Chinese Medical Association; CT, conventional therapy; AA, acupoint application; Acu, acupuncture; Moxibustion, moxibustion; AI, acupoint injection; MT, massage therapy; Cupping, cupping; NI, non-invasive therapy; NK, needle-knife therapy; CSTP, core stability training program; IH, subcutaneous injection; TENS, transcutaneous electrical nerve stimulation. ①BASDAI, Bath Ankylosing Spondylitis Disease Activity Index; ②BASFI, Bath Ankylosing Spondylitis Functional Index; ③VAS, Visual Analogue Scale pain score; ④CRP, C-reactive protein; ⑤ESR, erythrocyte sedimentation rate.

References:

1.	Chen Zhanglian & Pang Gensheng. Clinical study of dynamic moxibustion-assisted acupuncture in treating early-to-mid-stage adult-onset ankylosing spondylitis. Chinese Journal of Acupuncture and Moxibustion 36 (2016).

2.	Dong Tiantian, Pang Yazheng, Li Jinling, Sun Chunquan & Yang Jiguo. Therapeutic Observation of Du Moxibustion in Alleviating Pain Symptoms and Its Effect on Thermal Imaging in Ankylosing Spondylitis. Sichuan Journal of Traditional Chinese Medicine 35 (2017).

3.	Gao Yan, Li Feixia, Guo Huiqing, Zhou Zipeng & Zhang Yishan. Clinical study on acupuncture therapy improving pain and functional mobility in patients with ankylosing spondylitis. Chinese Journal of Rehabilitation Medicine 21 (2013).

4.	Huang Yongjie. Randomised parallel-group study of acupuncture treatment for ankylosing spondylitis. Journal of Practical Internal Medicine of Traditional Chinese Medicine (2016).

5.	Jin Haipeng, Wu Qiuyan, Zhang Wei, Chen Shaomei & Chen Jinchun. Randomised parallel-group study of combined phlegm-resolving and blood-activating formula acupoint plaster application with Western medicine for spinal mobility limitation in ankylosing spondylitis. Journal of Practical Internal Medicine (2013).

6.	Li Wanggui, Tu Qian & Gu Xuejuan. Treatment of Early Ankylosing Spondylitis Primarily Using Moxibustion on the Governor Vessel and Its Effect on Serum Inflammatory Cytokine Levels in Patients. Chinese Journal of Acupuncture and Moxibustion (2010).

7.	Qiang Tianming, Yan A. Li A. Treatment of 60 Cases of Ankylosing Spondylitis Using Acupuncture Knife. Western Chinese Medicine 26 (2013).

8.	Wang Mingming & Cai Shengchao. Clinical efficacy observation of acupuncture with "dragon bone needles" combined with Tongmai Wenyang moxibustion for ankylosing spondylitis. Journal of Shanxi University of Traditional Chinese Medicine 17 (2016).

9.	Xie, Yuying & Xue, Luan. Observation on the Therapeutic Effect of Acupoint Plaster Application Combined with Direct Current in the Treatment of Ankylosing Spondylitis. Shanghai Journal of Acupuncture and Moxibustion 34, 775–778 (2015). https://doi.org/10.13460/j.issn.1005-0957.2015.08.0775

10.	Yang Xiyun, Guo Jian, Chen Zhiru & Zhao Dacheng. Effects of acupuncture knife therapy on P-substance, IL-6, IL-2, and TNF-α expression in patients with ankylosing spondylitis. Chinese Journal of Basic Medicine (2015).

11.	Yue Yue & Gao Mingli. Combined application of analgesic acupoint patches and sulfasalazine in treating kidney deficiency with cold stagnation in ankylosing spondylitis. Journal of Practical Internal Medicine of Traditional Chinese Medicine (2014).

12.	Zhang Mengyu, Song Yongwei & Li Yang. Clinical observation on the combined treatment of ginger-separated moxibustion on the Governor Vessel, functional exercises and Western medication for ankylosing spondylitis. Rheumatology and Arthritis 5 (2016).

13.	Chen Yunjie et al. Clinical observation on the combined treatment of acupoint injection with medical ozone water and sacroiliac joint injection for ankylosing spondylitis. Guangming Traditional Chinese Medicine (2020).

14.	Deng Guiyi et al. Effects of Acupuncture Combined with Chiropractic Manipulation on Physical Function and Biochemical Indicators in Patients with Ankylosing Spondylitis. Journal of Acupuncture and Massage Medicine (English Edition) 19, 206–212 (2021).

15.	Guo Dajiang, Wang Caiyue, Yin Zhilong, Wu Huili & Dong Chunhua. Treatment of 45 Cases of Kidney Deficiency with Du Meridian Cold Pattern Ankylosing Spondylitis Using Du Meridian Moxibustion Therapy. Modern Distance Education in Chinese Medicine 19 (2021).

16.	He Lietao, Zou Zhuocheng & Cai Huiqian. Clinical Observation on the Efficacy of Kidney-Nourishing and Du Meridian-Regulating Acupuncture in Treating Ankylosing Spondylitis. Guangxi Journal of Traditional Chinese Medicine 43 (2020).

17.	Hu Zhufang, Wan Chengxian, Li Xiaoyun & Tu Yan. Clinical study on acupoint plaster application for ankylosing spondylitis. Practical Clinical Medicine 22, 75–76,85 (2021). https://doi.org/10.13764/j.cnki.lcsy. 2021.06.023

18.	Li Zhi, Chen Huibing & Wen Xingliang. The effect of moxibustion on the Du Meridian on the quality of life of patients with kidney yang deficiency-type ankylosing spondylitis. Chinese and Foreign Medical Care 39 (2020).

19. Ren Yingjie, Li Feng, Yan Jun, Wan Chao & Ma Junjie. Clinical Observation on Modified Du Meridian Moxibustion Combined with Fire Needle Therapy for Kidney Deficiency with Cold-Damp Pattern in Ankylosing Spondylitis. Hebei Journal of Traditional Chinese Medicine 42 (2020).

20.	Song Hongyan & Li Xia. Research on the Efficacy of Traditional Chinese Medicine Acupuncture in Treating Ankylosing Spondylitis. Clinical Research in Traditional Chinese Medicine 9 (2017).

21.	Zuo Zheng, Liu Zili, Yuan Kai, Wang Yaoling & Dong Kangli. Efficacy and Mechanism of Changshe Moxibustion in Treating Ankylosing Spondylitis Based on Th17/Treg/Th1 Immune Imbalance. Chinese Journal of Acupuncture and Moxibustion 38 (2018).

22.	Gao Ping, Wu Xiaoying, Guo Suwen, Wang Haoquan & Liu Shuyi. Clinical efficacy of warming-yang cupping combined with fire-dragon moxibustion in treating kidney deficiency with督寒 pattern in ankylosing spondylitis. Wisdom Health 11 (2025).

23.	Li Chongxin & Wang Yong. Analysis of the efficacy of electronic moxibustion in treating ankylosing spondylitis. Wisdom Health 9 (2023).

24.	Li Na, Yuan Qian & Zhang Jie. Clinical observation of wandering cupping on the bladder meridian combined with Du moxibustion for treating kidney deficiency with Du cold pattern in ankylosing spondylitis. Journal of Liaoning University of Traditional Chinese Medicine 23 (2021).

25. Ma Yao et al. Acupuncture combined with Du moxibustion for treating early-to-mid stage ankylosing spondylitis and its effect on sacroiliac joint bone marrow oedema. Chinese Acupuncture and Moxibustion 42 (2022).

26.	Rong Xiaohong & Liu Guixiang. Effects of Moxibustion on Rehabilitation Outcomes and Quality of Life in Patients with Ankylosing Spondylitis. Reflex Therapy and Rehabilitation Medicine 4 (2023).

27.	Teng Jinyan et al. Clinical observation on the efficacy of Du meridian moxibustion combined with kidney-tonifying and stasis-resolving acupuncture in treating acute flare-ups of ankylosing spondylitis. Journal of Acupuncture and Moxibustion 37, 22–26 (2021). https://doi.org/10.19917/j.cnki.1005-0779.021049

28.	Wang Wanyun, Zhao Chen, Zhang Min, Zhang Jingjing & Yu Hongwu. Clinical observation of Li Ji acupuncture combined with Du moxibustion in treating kidney deficiency with Du cold pattern in ankylosing spondylitis. Chinese Journal of Clinical Medicine 14 (2022).

29.	Wang Xinting, Zheng Shili, Huang Haiqiong, Fang Fang & Zhou Taimin. Effects of Warm Acupuncture Combined with Spinal Fixed-Point Realignment on Trunk Function and Inflammatory Factors in Patients with Ankylosing Spondylitis. Modern Practical Medicine 34 (2022).

30.	Zhang Yiting, Liu Qiuhong, Li Xiaoru, Zhao Lin & Qian Yiling. Clinical observation of Sanfu plasters combined with celecoxib in treating 25 cases of kidney deficiency with Du cold pattern ankylosing spondylitis. Rheumatology and Arthritis 10 (2021).

31. Zhou Xiaoli, Qiao Pingping, Zhang Jian, Guo Hailong & Qin Wenjun. A study on OPG/RANKL regulation in patients with ankylosing spondylitis and reduced bone mass or osteoporosis treated with acupuncture and medication. Rheumatology and Arthritis 10 (2021).

**Table S3.2.** Abbreviations for Interventions and Outcome Indicators

| Classifications | Full name | Abbreviations |
| --- | --- | --- |
|  | Conventional therapy | CT |
|  | Acupoint application | AA |
|  | Acupuncture | Acu |
|  | Moxibustion | Moxibustion |
|  | Acupoint injection | AI |
|  | Massage therapy | MT |
| All treatment modalities | Cupping | Cupping |
|  | Non-invasive treatment | NI |
|  | Needle knife | NK |
|  | Core Stability Training Programme | CSTP |
|  | Subcutaneous injection | IH |
|  | Transcutaneous electrical nerve stimulation | TENS |
|  | Bath Ankylosing Spondylitis Disease Activity Index | BASDAI |
|  | BathAnkylosing Spondylitis Functional Index | BASFI |
| Outcome indicators | Visual analogue scale | VAS |
|  | C-reactive protein | CRP |
|  | Erythrocyte sedimentation rate | ESR |

**Appendix 4. Risk of Bias of Randomized Clinical Trials**

**Appendix 5. League Table**

**Table S5.1.** League Table of BASDAI.

| AA |  |  |  |  |  |  |  |  |  |
| --- | --- | --- | --- | --- | --- | --- | --- | --- | --- |
| -0.76 (-1.65,0.12) | Acu+Moxibustion |  |  |  |  |  |  |  |  |
| -0.94 (-2.04,0.16) | -0.18 (-1.18,0.82) | NK |  |  |  |  |  |  |  |
| -1.05 (-2.07,-0.03) | -0.29 (-1.08,0.50) | -0.11 (-1.23,1.01) | Moxibustion+Cupping |  |  |  |  |  |  |
| -1.14 (-2.03,-0.25) | -0.38 (-1.04,0.28) | -0.20 (-1.19,0.80) | -0.09 (-0.97,0.79) | Acu+MT |  |  |  |  |  |
| -1.51 (-2.58,-0.44) | -0.74 (-1.49,0.00) | -0.56 (-1.72,0.60) | -0.45 (-1.16,0.25) | -0.37 (-1.28,0.55) | Moxibustion |  |  |  |  |
| -1.63 (-2.85,-0.41) | -0.87 (-1.70,-0.03) | -0.69 (-1.99,0.61) | -0.58 (-1.73,0.58) | -0.49 (-1.55,0.57) | -0.12 (-1.25,1.00) | Acu |  |  |  |
| -1.81 (-2.90,-0.73) | -1.05 (-2.03,-0.07) | -0.87 (-2.04,0.30) | -0.76 (-1.86,0.34) | -0.67 (-1.65,0.31) | -0.31 (-1.45,0.84) | -0.18 (-1.47,1.10) | AI |  |  |
| -1.97 (-2.68,-1.26) | -1.21 (-1.74,-0.68) | -1.03 (-1.87,-0.19) | -0.92 (-1.65,-0.19) | -0.83 (-1.37,-0.30) | -0.47 (-1.27,0.33) | -0.34 (-1.34,0.65) | -0.16 (-0.98,0.66) | CT |  |
| -2.30 (-3.28,-1.32) | -1.54 (-2.18,-0.90) | -1.36 (-2.43,-0.28) | -1.25 (-2.19,-0.31) | -1.16 (-1.77,-0.55) | -0.79 (-1.73,0.15) | -0.67 (-1.72,0.38) | -0.49 (-1.55,0.58) | -0.33 (-1.00,0.35) | IH |

Note: CT, conventional therapy; AA, acupoint application; Acu, acupuncture; Moxibustion, moxibustion; AI, acupoint injection; MT, massage therapy; Cupping, cupping; NI, non-invasive therapy; NK, needle-knife therapy; CSTP, core stability training program; IH, subcutaneous injection; TENS, transcutaneous electrical nerve stimulation.

**Table S5.2.** League Table of BASFI.

| Acu |  |  |  |  |  |  |  |  |  |
| --- | --- | --- | --- | --- | --- | --- | --- | --- | --- |
| -0.53 (-7.55,6.50) | Moxibustion+Cupping |  |  |  |  |  |  |  |  |
| -2.23 (-8.55,4.10) | -1.70 (-6.91,3.51) | Moxibustion |  |  |  |  |  |  |  |
| -2.63 (-6.64,1.39) | -2.10 (-8.38,4.19) | -0.40 (-5.60,4.80) | Acu+Moxibustion |  |  |  |  |  |  |
| -3.99 (-10.88,2.90) | -3.46 (-12.11,5.18) | -1.76 (-9.94,6.41) | -1.37 (-8.04,5.31) | NK |  |  |  |  |  |
| -4.03 (-10.92,2.86) | -3.50 (-12.15,5.14) | -1.80 (-9.98,6.38) | -1.41 (-8.08,5.27) | -0.04 (-8.35,8.27) | AA |  |  |  |  |
| -4.14 (-9.50,1.22) | -3.61 (-10.98,3.75) | -1.92 (-8.55,4.72) | -1.52 (-5.92,2.89) | -0.15 (-7.47,7.17) | -0.11 (-7.43,7.21) | IH |  |  |  |
| -4.15 (-8.77,0.46) | -3.62 (-10.57,3.32) | -1.93 (-8.24,4.39) | -1.53 (-5.67,2.61) | -0.16 (-6.75,6.43) | -0.12 (-6.71,6.47) | -0.01 (-4.56,4.53) | Acu+MT |  |  |
| -4.46 (-11.35,2.43) | -3.93 (-12.58,4.72) | -2.23 (-10.41,5.95) | -1.84 (-8.51,4.84) | -0.47 (-8.78,7.84) | -0.43 (-8.74,7.88) | -0.32 (-7.64,7.01) | -0.31 (-6.90,6.28) | AI |  |
| -4.89 (-8.50,-1.29) | -4.36 (-10.71,1.98) | -2.66 (-8.36,3.03) | -2.27 (-5.44,0.91) | -0.90 (-6.77,4.97) | -0.86 (-6.73,5.01) | -0.75 (-5.12,3.62) | -0.74 (-3.73,2.25) | -0.43 (-6.31,5.45) | CT |

Note: CT, conventional therapy; AA, acupoint application; Acu, acupuncture; Moxibustion, moxibustion; AI, acupoint injection; MT, massage therapy; Cupping, cupping; NI, non-invasive therapy; NK, needle-knife therapy; CSTP, core stability training program; IH, subcutaneous injection; TENS, transcutaneous electrical nerve stimulation.

**Table S5.3.** League Table of VAS.

| Moxibustion+Cupping |  |  |  |  |  |  |  |  |  |
| --- | --- | --- | --- | --- | --- | --- | --- | --- | --- |
| -0.47 (-1.29,0.36) | Acu+Moxibustion |  |  |  |  |  |  |  |  |
| -0.77 (-1.82,0.29) | -0.30 (-1.22,0.62) | Acu+MT |  |  |  |  |  |  |  |
| -0.76 (-1.46,-0.06) | -0.29 (-1.01,0.43) | 0.01 (-0.97,0.99) | Moxibustion |  |  |  |  |  |  |
| -1.05 (-2.03,-0.07) | -0.59 (-1.31,0.13) | -0.29 (-1.35,0.77) | -0.29 (-1.19,0.60) | Acu |  |  |  |  |  |
| -1.37 (-2.46,-0.28) | -0.91 (-1.86,0.05) | -0.61 (-1.77,0.56) | -0.61 (-1.63,0.40) | -0.32 (-1.41,0.77) | AA |  |  |  |  |
| -1.75 (-2.98,-0.52) | -1.28 (-2.39,-0.17) | -0.98 (-1.61,-0.35) | -0.99 (-2.15,0.18) | -0.70 (-1.93,0.54) | -0.37 (-1.70,0.95) | IH |  |  |  |
| -1.88 (-2.57,-1.19) | -1.42 (-1.86,-0.97) | -1.12 (-1.92,-0.32) | -1.12 (-1.69,-0.56) | -0.83 (-1.52,-0.14) | -0.51 (-1.35,0.33) | -0.14 (-1.15,0.88) | CT |  |  |
| -1.94 (-3.06,-0.82) | -1.48 (-2.47,-0.48) | -1.18 (-2.37,0.02) | -1.18 (-2.23,-0.13) | -0.89 (-2.02,0.23) | -0.57 (-1.79,0.65) | -0.20 (-1.55,1.16) | -0.06 (-0.95,0.83) | AI |  |
| -2.62 (-4.00,-1.23) | -2.15 (-3.44,-0.86) | -1.85 (-2.75,-0.95) | -1.86 (-3.19,-0.53) | -1.56 (-2.96,-0.17) | -1.24 (-2.71,0.23) | -0.87 (-1.97,0.23) | -0.73 (-1.94,0.47) | -0.67 (-2.17,0.82) | TENS |

Note: CT, conventional therapy; AA, acupoint application; Acu, acupuncture; Moxibustion, moxibustion; AI, acupoint injection; MT, massage therapy; Cupping, cupping; NI, non-invasive therapy; NK, needle-knife therapy; CSTP, core stability training program; IH, subcutaneous injection; TENS, transcutaneous electrical nerve stimulation.

**Table S5.4.** League Table of CRP.

| Acu |  |  |  |  |  |  |  |  |  |  |
| --- | --- | --- | --- | --- | --- | --- | --- | --- | --- | --- |
| -28.76 (-59.46,1.94) | Acu+Moxibustion |  |  |  |  |  |  |  |  |  |
| -40.14 (-82.90,2.61) | -11.38 (-47.47,24.71) | Moxibustion+Cupping |  |  |  |  |  |  |  |  |
| -40.69 (-76.83,-4.55) | -11.93 (-38.62,14.76) | -0.55 (-31.21,30.12) | Moxibustion |  |  |  |  |  |  |  |
| -44.34 (-85.94,-2.74) | -15.58 (-51.28,20.13) | -4.19 (-45.65,37.27) | -3.65 (-39.27,31.98) | AI |  |  |  |  |  |  |
| -45.14 (-95.28,5.00) | -16.38 (-61.74,28.99) | -4.99 (-55.02,45.03) | -4.45 (-49.75,40.86) | -0.80 (-49.09,47.49) | CSTP |  |  |  |  |  |
| -46.28 (-96.40,3.85) | -17.52 (-62.87,27.83) | -6.13 (-56.14,43.88) | -5.59 (-50.88,39.70) | -1.94 (-50.22,46.33) | -1.14 (-56.94,54.66) | AA |  |  |  |  |
| -46.91 (-86.96,-6.87) | -18.15 (-52.03,15.72) | -6.77 (-46.67,33.13) | -6.22 (-40.01,27.57) | -2.58 (-40.28,35.12) | -1.78 (-48.73,45.18) | -0.64 (-47.58,46.30) | Acu+MT |  |  |  |
| -50.09 (-102.89,2.71) | -21.33 (-69.62,26.96) | -9.95 (-62.63,42.74) | -9.40 (-57.63,38.83) | -5.75 (-56.80,45.29) | -4.95 (-63.17,53.26) | -3.81 (-62.01,54.39) | -3.18 (-40.93,34.57) | IH |  |  |
| -48.24 (-79.16,-17.31) | -19.48 (-41.85,2.89) | -8.09 (-38.83,22.64) | -7.55 (-29.79,14.70) | -3.90 (-31.73,23.92) | -3.10 (-42.57,36.37) | -1.96 (-41.41,37.49) | -1.32 (-26.76,24.11) | 1.85 (-40.94,44.64) | CT |  |
| -51.72 (-107.72,4.28) | -22.96 (-74.73,28.81) | -11.58 (-67.48,44.32) | -11.03 (-62.75,40.68) | -7.39 (-61.74,46.96) | -6.59 (-67.72,54.55) | -5.45 (-66.57,55.68) | -4.81 (-44.11,34.49) | -1.63 (-56.11,52.84) | -3.49 (-50.17,43.20) | TENS |

Note: CT, conventional therapy; AA, acupoint application; Acu, acupuncture; Moxibustion, moxibustion; AI, acupoint injection; MT, massage therapy; Cupping, cupping; NI, non-invasive therapy; NK, needle-knife therapy; CSTP, core stability training program; IH, subcutaneous injection; TENS, transcutaneous electrical nerve stimulation.

**Table S5.5.** League Table of ESR.

| Acu |  |  |  |  |  |  |  |  |  |
| --- | --- | --- | --- | --- | --- | --- | --- | --- | --- |
| -3.63 (-13.28,6.02) | Acu+Moxibustion |  |  |  |  |  |  |  |  |
| -4.81 (-17.27,7.64) | -1.18 (-10.20,7.84) | Moxibustion+Cupping |  |  |  |  |  |  |  |
| -6.82 (-21.32,7.67) | -3.19 (-14.94,8.56) | -2.01 (-14.58,10.56) | AI |  |  |  |  |  |  |
| -8.85 (-23.81,6.11) | -5.22 (-17.54,7.10) | -4.04 (-17.15,9.07) | -2.03 (-17.04,12.98) | CSTP |  |  |  |  |  |
| -9.31 (-20.37,1.75) | -5.68 (-12.50,1.14) | -4.50 (-12.31,3.31) | -2.49 (-13.90,8.92) | -0.46 (-12.46,11.54) | Moxibustion |  |  |  |  |
| -11.78 (-23.72,0.16) | -8.15 (-16.53,0.24) | -6.96 (-16.47,2.54) | -4.95 (-16.95,7.04) | -2.92 (-15.47,9.63) | -2.47 (-10.37,5.44) | Acu+MT |  |  |  |
| -14.35 (-24.57,-4.14) | -10.72 (-16.40,-5.04) | -9.54 (-16.77,-2.31) | -7.53 (-17.82,2.76) | -5.50 (-16.43,5.43) | -5.04 (-9.99,-0.10) | -2.58 (-8.74,3.59) | CT |  |  |
| -16.38 (-32.04,-0.72) | -12.75 (-25.90,0.40) | -11.57 (-25.46,2.32) | -9.56 (-25.26,6.14) | -7.53 (-23.66,8.60) | -7.07 (-19.92,5.78) | -4.60 (-14.77,5.56) | -2.03 (-13.89,9.83) | IH |  |
| -18.51 (-34.78,-2.23) | -14.88 (-28.76,-1.00) | -13.69 (-28.28,0.89) | -11.68 (-28.00,4.63) | -9.65 (-26.38,7.07) | -9.20 (-22.79,4.40) | -6.73 (-17.79,4.33) | -4.15 (-16.82,8.51) | -2.13 (-17.15,12.90) | TENS |

Note: CT, conventional therapy; AA, acupoint application; Acu, acupuncture; Moxibustion, moxibustion; AI, acupoint injection; MT, massage therapy; Cupping, cupping; NI, non-invasive therapy; NK, needle-knife therapy; CSTP, core stability training program; IH, subcutaneous injection; TENS, transcutaneous electrical nerve stimulation.

**Appendix 6. GRADE**

**Table S6.1.** The Grading of Recommendations Assessment, Development and Evaluation (GRADE) assessment for BASDAI.

| Comparison | N of studies | Within-study bias | Reporting bias | Indirectness | Imprecision | Heterogeneity | Incoherence | Confidence rating |
| --- | --- | --- | --- | --- | --- | --- | --- | --- |
| CT:AA | 2 | Some concerns | Low risk | No concerns | Some concerns | Some concerns | Major concerns | Very Low |
| CT:Acu | 1 | Major concerns | Low risk | No concerns | Major concerns | No concerns | No concerns | Low |
| CT:AI | 1 | Major concerns | Low risk | No concerns | Major concerns | No concerns | Major concerns | Low |
| CT:NK | 1 | Major concerns | Low risk | No concerns | Some concerns | Some concerns | Major concerns | Low |
| CT:Acu+Moxibustion | 2 | Major concerns | Low risk | No concerns | Some concerns | Some concerns | Major concerns | Low |
| CT:Moxibustion+Cupping | 1 | Some concerns | Low risk | No concerns | Some concerns | Some concerns | No concerns | Very Low |
| CT:Acu+MT | 2 | Major concerns | Low risk | No concerns | No concerns | Some concerns | Some concerns | Low |
| IH:Acu+Moxibustion | 1 | Major concerns | Low risk | No concerns | No concerns | No concerns | No concerns | Moderate |
| IH:Acu+MT | 1 | Major concerns | Low risk | No concerns | No concerns | No concerns | Some concerns | Low |
| Acu+Moxibustion:Acu | 1 | Major concerns | Low risk | No concerns | Some concerns | Some concerns | No concerns | Very Low |
| Acu+Moxibustion:Moxibustion | 1 | Major concerns | Low risk | No concerns | Some concerns | Some concerns | No concerns | Moderate |
| Moxibustion+Cupping:Moxibustion | 1 | Major concerns | Low risk | No concerns | Some concerns | Some concerns | No concerns | Low |
| CT:Moxibustion | 0 | Major concerns | Low risk | No concerns | Major concerns | No concerns | Major concerns | Low |
| CT:IH | 0 | Major concerns | Low risk | No concerns | No concerns | No concerns | Major concerns | Very Low |
| AA:Acu | 0 | Major concerns | Low risk | No concerns | Some concerns | Some concerns | Major concerns | Low |
| AA:Moxibustion | 0 | Major concerns | Low risk | No concerns | Some concerns | Some concerns | Major concerns | Very Low |
| AA:AI | 0 | Major concerns | Low risk | No concerns | Some concerns | Some concerns | Major concerns | Low |
| AA:NK | 0 | Major concerns | Low risk | No concerns | Major concerns | No concerns | Major concerns | Moderate |
| IH:AA | 0 | Major concerns | Low risk | No concerns | No concerns | No concerns | Major concerns | Moderate |
| Acu+Moxibustion:AA | 0 | Major concerns | Low risk | No concerns | Some concerns | Some concerns | Major concerns | Very Low |
| Moxibustion+Cupping:AA | 0 | Some concerns | Low risk | No concerns | Major concerns | No concerns | Major concerns | Low |
| Acu+MT:AA | 0 | Major concerns | Low risk | No concerns | Some concerns | Some concerns | Major concerns | Very Low |
| Acu:Moxibustion | 0 | Major concerns | Low risk | No concerns | Major concerns | No concerns | Major concerns | Low |
| Acu:AI | 0 | Major concerns | Low risk | No concerns | Major concerns | No concerns | Major concerns | Very Low |
| Acu:NK | 0 | Major concerns | Low risk | No concerns | Some concerns | Some concerns | Major concerns | Moderate |
| IH:Acu | 0 | Major concerns | Low risk | No concerns | No concerns | No concerns | Major concerns | Very Low |
| Moxibustion+Cupping:Acu | 0 | Major concerns | Low risk | No concerns | Some concerns | Some concerns | Major concerns | Low |
| Acu+MT:Acu | 0 | Major concerns | Low risk | No concerns | No concerns | Some concerns | Major concerns | Very Low |
| Moxibustion:AI | 0 | Major concerns | Low risk | No concerns | Major concerns | No concerns | Major concerns | Low |
| Moxibustion:NK | 0 | Major concerns | Low risk | No concerns | Some concerns | Some concerns | Major concerns | Low |
| IH:Moxibustion | 0 | Major concerns | Low risk | No concerns | No concerns | No concerns | Major concerns | Low |
| Acu+MT:Moxibustion | 0 | Major concerns | Low risk | No concerns | No concerns | Some concerns | Major concerns | Moderate |
| AI:NK | 0 | Major concerns | Low risk | No concerns | Major concerns | No concerns | Major concerns | Low |
| IH:AI | 0 | Major concerns | Low risk | No concerns | No concerns | No concerns | Major concerns | Low |
| Acu+Moxibustion:AI | 0 | Major concerns | Low risk | No concerns | Major concerns | No concerns | Major concerns | Very Low |
| Moxibustion+Cupping:AI | 0 | Major concerns | Low risk | No concerns | Major concerns | No concerns | Major concerns | Low |
| Acu+MT:AI | 0 | Major concerns | Low risk | No concerns | Some concerns | Some concerns | Major concerns | Low |
| IH:NK | 0 | Major concerns | Low risk | No concerns | No concerns | No concerns | Major concerns | Very Low |
| Acu+Moxibustion:NK | 0 | Major concerns | Low risk | No concerns | Major concerns | No concerns | Major concerns | Moderate |
| Moxibustion+Cupping:NK | 0 | Major concerns | Low risk | No concerns | Major concerns | No concerns | Major concerns | Low |
| Acu+MT:NK | 0 | Major concerns | Low risk | No concerns | Major concerns | No concerns | Major concerns | Very Low |
| IH:Moxibustion+Cupping | 0 | Major concerns | Low risk | No concerns | No concerns | No concerns | Major concerns | Low |
| Acu+Moxibustion:Moxibustion+Cupping | 0 | Major concerns | Low risk | No concerns | Major concerns | No concerns | Major concerns | Low |
| Acu+Moxibustion:Acu+MT | 0 | Major concerns | Low risk | No concerns | No concerns | Some concerns | Major concerns | Low |
| Moxibustion+Cupping:Acu+MT | 0 | Major concerns | Low risk | No concerns | Some concerns | Some concerns | Major concerns | Very Low |

**Table S6.2.** The Grading of Recommendations Assessment, Development and Evaluation (GRADE) assessment for BASFI.

| Comparison | N of studies | Within-study bias | Reporting bias | Indirectness | Imprecision | Heterogeneity | Incoherence | Confidence rating |
| --- | --- | --- | --- | --- | --- | --- | --- | --- |
| CT:AA | 2 | Some concerns | Low risk | No concerns | Some concerns | Some concerns | Major concerns | Low |
| CT:Acu | 1 | Major concerns | Low risk | No concerns | Major concerns | No concerns | No concerns | Low |
| CT:AI | 1 | Major concerns | Low risk | No concerns | Major concerns | No concerns | Major concerns | Moderate |
| CT:NK | 1 | Major concerns | Low risk | No concerns | Some concerns | Some concerns | Major concerns | Low |
| CT:Acu+Moxibustion | 2 | Major concerns | Low risk | No concerns | Some concerns | Some concerns | Some concerns | Moderate |
| CT:Moxibustion+Cupping | 1 | Some concerns | Low risk | No concerns | Some concerns | Some concerns | No concerns | Low |
| CT:Acu+MT | 2 | Major concerns | Low risk | No concerns | No concerns | Some concerns | Some concerns | Very Low |
| IH:Acu+Moxibustion | 1 | Major concerns | Low risk | No concerns | No concerns | No concerns | No concerns | Low |
| IH:Acu+MT | 1 | Major concerns | Low risk | No concerns | No concerns | No concerns | No concerns | Very Low |
| Acu+Moxibustion:Acu | 1 | Major concerns | Low risk | No concerns | Some concerns | Some concerns | No concerns | Low |
| Acu+Moxibustion:Moxibustion | 1 | Major concerns | Low risk | No concerns | Major concerns | No concerns | No concerns | Very Low |
| Moxibustion+Cupping:Moxibustion | 1 | Major concerns | Low risk | No concerns | Some concerns | Some concerns | No concerns | Moderate |
| CT:Moxibustion | 0 | Major concerns | Low risk | No concerns | Major concerns | No concerns | Major concerns | Moderate |
| CT:IH | 0 | Major concerns | Low risk | No concerns | No concerns | No concerns | Major concerns | Low |
| AA:Acu | 0 | Major concerns | Low risk | No concerns | Some concerns | Some concerns | Major concerns | Very Low |
| AA:Moxibustion | 0 | Major concerns | Low risk | No concerns | Some concerns | Some concerns | Major concerns | Moderate |
| AA:AI | 0 | Major concerns | Low risk | No concerns | Major concerns | No concerns | Major concerns | Very Low |
| AA:NK | 0 | Major concerns | Low risk | No concerns | Major concerns | No concerns | Major concerns | Low |
| IH:AA | 0 | Major concerns | Low risk | No concerns | No concerns | No concerns | Major concerns | Low |
| Acu+Moxibustion:AA | 0 | Major concerns | Low risk | No concerns | Some concerns | Some concerns | Major concerns | Low |
| Moxibustion+Cupping:AA | 0 | Some concerns | Low risk | No concerns | Major concerns | No concerns | Major concerns | Low |
| Acu+MT:AA | 0 | Major concerns | Low risk | No concerns | Some concerns | Some concerns | Major concerns | Very Low |
| Acu:Moxibustion | 0 | Major concerns | Low risk | No concerns | Major concerns | No concerns | Major concerns | Moderate |
| Acu:AI | 0 | Major concerns | Low risk | No concerns | Major concerns | No concerns | Major concerns | Low |
| Acu:NK | 0 | Major concerns | Low risk | No concerns | Major concerns | No concerns | Major concerns | Very Low |
| IH:Acu | 0 | Major concerns | Low risk | No concerns | No concerns | No concerns | Major concerns | Very Low |
| Moxibustion+Cupping:Acu | 0 | Major concerns | Low risk | No concerns | Major concerns | No concerns | Major concerns | Low |
| Acu+MT:Acu | 0 | Major concerns | Low risk | No concerns | No concerns | Major concerns | Major concerns | Low |
| Moxibustion:AI | 0 | Major concerns | Low risk | No concerns | Major concerns | No concerns | Major concerns | Low |
| Moxibustion:NK | 0 | Major concerns | Low risk | No concerns | Major concerns | No concerns | Major concerns | Low |
| IH:Moxibustion | 0 | Major concerns | Low risk | No concerns | No concerns | No concerns | Major concerns | Moderate |
| Acu+MT:Moxibustion | 0 | Major concerns | Low risk | No concerns | No concerns | Major concerns | Major concerns | Very Low |
| AI:NK | 0 | Major concerns | Low risk | No concerns | Major concerns | No concerns | Major concerns | Moderate |
| IH:AI | 0 | Major concerns | Low risk | No concerns | No concerns | No concerns | Major concerns | Low |
| Acu+Moxibustion:AI | 0 | Major concerns | Low risk | No concerns | Major concerns | No concerns | Major concerns | Low |
| Moxibustion+Cupping:AI | 0 | Major concerns | Low risk | No concerns | Major concerns | No concerns | Major concerns | Moderate |
| Acu+MT:AI | 0 | Major concerns | Low risk | No concerns | Some concerns | Some concerns | Major concerns | Very Low |
| IH:NK | 0 | Major concerns | Low risk | No concerns | No concerns | No concerns | Major concerns | Moderate |
| Acu+Moxibustion:NK | 0 | Major concerns | Low risk | No concerns | Major concerns | No concerns | Major concerns | Low |
| Moxibustion+Cupping:NK | 0 | Major concerns | Low risk | No concerns | Major concerns | No concerns | Major concerns | Low |
| Acu+MT:NK | 0 | Major concerns | Low risk | No concerns | Major concerns | No concerns | Major concerns | Very Low |
| IH:Moxibustion+Cupping | 0 | Major concerns | Low risk | No concerns | No concerns | No concerns | Major concerns | Moderate |
| Acu+Moxibustion:Moxibustion+Cupping | 0 | Major concerns | Low risk | No concerns | Major concerns | No concerns | Major concerns | Low |
| Acu+Moxibustion:Acu+MT | 0 | Major concerns | Low risk | No concerns | No concerns | Major concerns | Major concerns | Very Low |
| Moxibustion+Cupping:Acu+MT | 0 | Major concerns | Low risk | No concerns | Some concerns | Some concerns | Major concerns | Very Low |

**Table S6.3.** The Grading of Recommendations Assessment, Development and Evaluation (GRADE) assessment for VAS.

| Comparison | N of studies | Within-study bias | Reporting bias | Indirectness | Imprecision | Heterogeneity | Incoherence | Confidence rating |
| --- | --- | --- | --- | --- | --- | --- | --- | --- |
| CT:AA | 1 | Major concerns | Low risk | No concerns | No concerns | Some concerns | No concerns | Very Low |
| CT:Acu | 1 | Major concerns | Low risk | No concerns | No concerns | No concerns | No concerns | Low |
| CT:Moxibustion | 2 | Major concerns | Low risk | No concerns | No concerns | No concerns | No concerns | Low |
| CT:AI | 1 | Major concerns | Low risk | No concerns | No concerns | Some concerns | No concerns | Low |
| CT:Acu+Moxibustion | 3 | Some concerns | Low risk | No concerns | No concerns | No concerns | No concerns | Very Low |
| CT:Moxibustion+Cupping | 1 | Some concerns | Low risk | No concerns | No concerns | No concerns | No concerns | Moderate |
| CT:Acu+MT | 1 | Major concerns | Low risk | No concerns | No concerns | No concerns | No concerns | Low |
| IH:Acu+MT | 1 | Major concerns | Low risk | No concerns | No concerns | No concerns | No concerns | Low |
| TENS:Acu+MT | 1 | No concerns | Low risk | No concerns | No concerns | No concerns | No concerns | Moderate |
| Acu+Moxibustion:Acu | 1 | Major concerns | Low risk | No concerns | No concerns | Some concerns | No concerns | Moderate |
| Moxibustion+Cupping:Moxibustion | 1 | Major concerns | Low risk | No concerns | No concerns | Some concerns | No concerns | Very Low |
| CT:IH | 0 | Major concerns | Low risk | No concerns | No concerns | No concerns | No concerns | Low |
| CT:TENS | 0 | Some concerns | Low risk | No concerns | Some concerns | No concerns | No concerns | Very Low |
| AA:Acu | 0 | Major concerns | Low risk | No concerns | Some concerns | No concerns | No concerns | Low |
| AA:Moxibustion | 0 | Major concerns | Low risk | No concerns | Some concerns | No concerns | No concerns | Moderate |
| AA:AI | 0 | Major concerns | Low risk | No concerns | Some concerns | No concerns | No concerns | Moderate |
| IH:AA | 0 | Major concerns | Low risk | No concerns | No concerns | No concerns | No concerns | Low |
| TENS:AA | 0 | Some concerns | Low risk | No concerns | No concerns | Some concerns | No concerns | Low |
| Acu+Moxibustion:AA | 0 | Major concerns | Low risk | No concerns | No concerns | No concerns | No concerns | Low |
| Moxibustion+Cupping:AA | 0 | Major concerns | Low risk | No concerns | No concerns | No concerns | No concerns | Low |
| Acu+MT:AA | 0 | Major concerns | Low risk | No concerns | Some concerns | No concerns | No concerns | Very Low |
| Acu:Moxibustion | 0 | Major concerns | Low risk | No concerns | No concerns | No concerns | No concerns | Moderate |
| Acu:AI | 0 | Major concerns | Low risk | No concerns | Some concerns | No concerns | No concerns | Very Low |
| IH:Acu | 0 | Major concerns | Low risk | No concerns | No concerns | No concerns | No concerns | Low |
| TENS:Acu | 0 | Some concerns | Low risk | No concerns | No concerns | No concerns | No concerns | Low |
| Moxibustion+Cupping:Acu | 0 | Major concerns | Low risk | No concerns | No concerns | Some concerns | No concerns | Very Low |
| Acu+MT:Acu | 0 | Major concerns | Low risk | No concerns | No concerns | Some concerns | No concerns | Moderate |
| Moxibustion:AI | 0 | Major concerns | Low risk | No concerns | No concerns | Some concerns | No concerns | Low |
| IH:Moxibustion | 0 | Major concerns | Low risk | No concerns | No concerns | No concerns | No concerns | Low |
| TENS:Moxibustion | 0 | Some concerns | Low risk | No concerns | No concerns | No concerns | No concerns | Low |
| Acu+Moxibustion:Moxibustion | 0 | Some concerns | Low risk | No concerns | No concerns | Some concerns | No concerns | Low |
| Acu+MT:Moxibustion | 0 | Major concerns | Low risk | No concerns | No concerns | No concerns | No concerns | Moderate |
| IH:AI | 0 | Major concerns | Low risk | No concerns | No concerns | No concerns | No concerns | Moderate |
| TENS:AI | 0 | Some concerns | Low risk | No concerns | Some concerns | No concerns | No concerns | Very Low |
| Acu+Moxibustion:AI | 0 | Major concerns | Low risk | No concerns | No concerns | No concerns | No concerns | Low |
| Moxibustion+Cupping:AI | 0 | Major concerns | Low risk | No concerns | No concerns | No concerns | No concerns | Low |
| Acu+MT:AI | 0 | Major concerns | Low risk | No concerns | Some concerns | No concerns | No concerns | Low |
| IH:TENS | 0 | Some concerns | Low risk | No concerns | No concerns | No concerns | No concerns | Very Low |
| IH:Acu+Moxibustion | 0 | Major concerns | Low risk | No concerns | No concerns | No concerns | No concerns | Very Low |
| IH:Moxibustion+Cupping | 0 | Major concerns | Low risk | No concerns | No concerns | No concerns | No concerns | Low |
| TENS:Acu+Moxibustion | 0 | Some concerns | Low risk | No concerns | No concerns | No concerns | No concerns | Moderate |
| TENS:Moxibustion+Cupping | 0 | Some concerns | Low risk | No concerns | No concerns | No concerns | No concerns | Low |
| Acu+Moxibustion:Moxibustion+Cupping | 0 | Some concerns | Low risk | No concerns | No concerns | Some concerns | No concerns | Low |
| Acu+Moxibustion:Acu+MT | 0 | Major concerns | Low risk | No concerns | No concerns | Some concerns | No concerns | Moderate |
| Moxibustion+Cupping:Acu+MT | 0 | Major concerns | Low risk | No concerns | Some concerns | No concerns | No concerns | Very Low |

**Appendix 7. RoB 2**

|  | Domain 1. Randomization process | | | | | Domain 2. Deviations from intended interventions | | | | | | | | | Domain 3. Mising outcome data | | | | | | Domain 4. Measurement of the outcome | | | | | | | Domain 5. Selection of the reported result | | | | | Domain 6. Overall Bias | |
| --- | --- | --- | --- | --- | --- | --- | --- | --- | --- | --- | --- | --- | --- | --- | --- | --- | --- | --- | --- | --- | --- | --- | --- | --- | --- | --- | --- | --- | --- | --- | --- | --- | --- | --- |
| Study ID | 1.1 | 1.2 | 1.3 | 1.0 Algorithm result | 1.0 Assessor's Judgement | 2.1 | 2.2 | 2.3 | 2.4 | 2.5 | 2.6 | 2.7 | 2.0 Algorithm result | 2.0 Assessor's Judgement | 3.1 | 3.2 | 3.3 | 3.4 | 3.0 Algorithm result | 3.0 Assessor's judgement | 4.1 | 4.2 | 4.3 | 4.4 | 4.5 | 4.0 Algorithm result | 4.0 Assessor's Judgement | 5.1 | 5.2 | 5.3 | 5.0 Algorithm result | 5.0 Assessor's Judgement | Algorithm's overall Judgement | Assessor's overall Judgement |
| Jin2013 | Y | PY | N | Low | Low | Y | Y | N | NA | NA | Y | NA | Low | Low | Y | NA | NA | NA | Low | Low | N | N | N | NA | NA | Low | Low | Y | N | N | Low | Low | Low | Low |
| Yue2014 | Y | PN | N | High | High | Y | Y | N | NA | NA | PY | NA | Low | Low | N | N | NI | NI | High | High | N | N | N | NA | NA | Low | Low | Y | N | N | Low | Low | High | High |
| Huang2016 | Y | PY | N | Low | Low | Y | Y | N | NA | NA | PY | NA | Low | Low | Y | NA | NA | NA | Low | Low | N | N | NI | NI | NI | High | High | NI | NI | NI | Some concerns | Some concerns | High | High |
| Ren2020 | Y | NI | N | Some concerns | Some concerns | Y | Y | N | NA | NA | NI | NI | High | High | NI | N | NI | NI | High | High | N | N | NI | NI | NI | High | High | NI | NI | NI | Some concerns | Some concerns | High | High |
| Rong2023 | Y | PY | N | Low | Low | Y | Y | N | NA | NA | Y | NA | Low | Low | Y | NA | NA | NA | Low | Low | N | N | N | NA | NA | Low | Low | Y | N | N | Low | Low | Low | Low |
| Chen2020 | Y | NI | N | Some concerns | Some concerns | Y | Y | N | NA | NA | PY | NA | Low | Low | PN | N | NI | NI | High | High | N | N | N | NA | NA | Low | Low | NI | NI | NI | Some concerns | Some concerns | High | High |
| Deng2021 | Y | NI | N | Some concerns | Some concerns | Y | Y | N | NA | NA | Y | NA | Low | Low | Y | NA | NA | NA | Low | Low | N | N | NI | NI | NI | High | High | NI | NI | NI | Some concerns | Some concerns | High | High |
| Dong2017 | Y | NI | N | Some concerns | Some concerns | Y | Y | N | NA | NA | Y | NA | Low | Low | Y | NA | NA | NA | Low | Low | N | N | N | NA | NA | Low | Low | NI | NI | NI | Some concerns | Some concerns | Some concerns | Some concerns |
| Gao2025 | Y | NI | N | Some concerns | Some concerns | Y | Y | N | NA | NA | NI | NI | High | High | NI | N | NI | NI | High | High | N | N | N | NA | NA | Low | Some concerns | NI | NI | NI | Some concerns | Some concerns | High | High |
| Gao2013 | Y | PY | N | Low | Low | Y | Y | N | NA | NA | Y | NA | Low | Low | Y | NA | NA | NA | Low | Low | N | N | N | NA | NA | Low | Low | Y | N | N | Low | Low | Low | Low |
| Guo2021 | Y | PY | N | Low | Low | Y | Y | N | NA | NA | NI | NI | High | High | Y | NA | NA | NA | Low | Low | N | N | NI | NI | NI | High | High | NI | NI | NI | Some concerns | Some concerns | High | High |
| He2020 | Y | NI | N | Some concerns | Some concerns | Y | Y | N | NA | NA | Y | NA | Low | Low | NI | N | NI | NI | High | High | N | N | NI | NI | NI | High | High | NI | NI | NI | Some concerns | Some concerns | High | High |
| Hu2021 | Y | NI | N | Some concerns | Some concerns | Y | Y | N | NA | NA | NI | NI | High | High | NI | N | NI | NI | High | High | N | N | NI | NI | NI | High | High | Y | N | N | Low | Low | High | High |
| Li2023 | Y | NI | N | Some concerns | Some concerns | Y | Y | N | NA | NA | NI | NI | High | High | Y | NA | NA | NA | Low | Low | N | N | NI | NI | NI | High | High | NI | NI | NI | Some concerns | Some concerns | High | High |
| Li2021 | Y | NI | N | Some concerns | Some concerns | Y | Y | N | NA | NA | Y | NA | Low | Low | Y | NA | NA | NA | Low | Low | N | N | N | NA | NA | Low | Low | NI | NI | NI | Some concerns | Some concerns | Some concerns | Some concerns |
| Li2020 | Y | NI | N | Some concerns | Some concerns | Y | Y | N | NA | NA | Y | NA | Low | Low | Y | NA | NA | NA | Low | Low | N | N | N | NA | NA | Low | Low | NI | NI | NI | Some concerns | Some concerns | Some concerns | Some concerns |
| Song2017 | Y | NI | N | Some concerns | Some concerns | Y | Y | N | NA | NA | NI | NI | High | High | Y | NA | NA | NA | Low | Low | N | N | NI | NI | NI | High | High | Y | N | N | Low | Low | High | High |
| Teng2021 | Y | PY | N | Low | Low | Y | Y | N | NA | NA | NI | NI | High | High | NI | N | NI | NI | High | High | N | N | N | NA | NA | Low | Low | NI | NI | NI | Some concerns | Some concerns | High | High |
| Wang2022 | Y | PY | N | Low | Low | Y | Y | N | NA | NA | Y | NA | Low | Low | NI | N | NI | NI | High | High | N | N | N | NA | NA | Low | Low | NI | NI | NI | Some concerns | Some concerns | High | High |
| Wang2016 | Y | NI | N | Some concerns | Some concerns | Y | Y | N | NA | NA | Y | NA | Low | Low | Y | NA | NA | NA | Low | Low | N | N | N | NA | NA | Low | Low | Y | N | N | Low | Low | Some concerns | Some concerns |
| Wang WY2022 | Y | Y | N | Low | Low | Y | Y | N | NA | NA | Y | NA | Low | Low | Y | NA | NA | NA | Low | Low | N | N | NI | NI | NI | High | High | NI | NI | NI | Some concerns | Some concerns | High | High |
| Yan2013 | Y | PY | N | Low | Low | Y | Y | N | NA | NA | NI | NI | High | High | NI | N | NI | NI | High | High | N | N | N | NA | NA | Low | Low | NI | NI | NI | Some concerns | Some concerns | High | High |
| Yang2015 | Y | NI | N | Some concerns | Some concerns | Y | Y | N | NA | NA | NI | NI | High | High | Y | NA | NA | NA | Low | Low | N | N | NI | NI | NI | High | High | Y | N | N | Low | Low | High | High |
| Zhang2016 | Y | NI | N | Some concerns | Some concerns | Y | Y | N | NA | NA | NI | NI | High | High | NI | N | NI | NI | High | High | N | N | N | NA | NA | Low | Low | NI | NI | NI | Some concerns | Some concerns | High | High |
| zhang2021 | Y | PY | N | Low | Low | Y | Y | N | NA | NA | Y | NA | Low | Low | Y | NA | NA | NA | Low | Low | N | N | N | NA | NA | Low | Low | Y | N | N | Low | Low | Low | Low |
| Zhou2021 | Y | NI | N | Some concerns | Some concerns | Y | Y | N | NA | NA | Y | NA | Low | Low | PN | N | PN | NA | Low | Low | N | N | NI | NI | NI | High | High | NI | NI | NI | Some concerns | Some concerns | High | High |
| Zuo2018 | Y | PY | N | Low | Low | Y | Y | N | NA | NA | PY | NA | Low | Low | Y | NA | NA | NA | Low | Low | N | N | N | NA | NA | Low | Low | Y | N | N | Low | Low | Low | Low |
| Chen2016 | Y | PY | N | Low | Low | Y | Y | N | NA | NA | NI | NI | High | High | Y | NA | NA | NA | Low | Low | N | N | NI | NI | NI | High | High | Y | N | N | Low | Low | High | High |
| Li2010 | Y | PN | N | High | High | Y | Y | N | NA | NA | Y | NA | Low | Low | Y | NA | NA | NA | Low | Low | N | N | N | NA | NA | Low | Low | NI | NI | NI | Some concerns | Some concerns | High | High |
| Ma2022 | Y | Y | N | Low | Low | Y | Y | N | NA | NA | PY | NA | Low | Low | PY | NA | NA | NA | Low | Low | N | N | Y | NI | NI | High | High | NI | NI | NI | Some concerns | Some concerns | High | High |
| Xie2015 | Y | NI | N | Some concerns | Some concerns | Y | Y | N | NA | NA | Y | NA | Low | Low | Y | NA | NA | NA | Low | Low | N | N | N | NA | NA | Low | Low | NI | NI | NI | Some concerns | Some concerns | Some concerns | Some concerns |
